# Effect of chromium (VI) toxicity on morpho-physiological characteristics, yield, and yield components of two chickpea (*Cicer arietinum* L.) varieties

**DOI:** 10.1371/journal.pone.0243032

**Published:** 2020-12-03

**Authors:** Deepti Singh, Nithi Lal Sharma, Chandan Kumar Singh, Susheel Kumar Sarkar, Ishwar Singh, Mohan Lal Dotaniya

**Affiliations:** 1 Department of Botany, Meerut College, Meerut, India; 2 Division of Genetics, ICAR-Indian Agricultural Research Institute, New Delhi, India; 3 Division of Design of Experiments (DE), ICAR-Indian Agricultural Statistics Research Institute, New Delhi, India; 4 Department of Botany, CCS University, Meerut, India; 5 ICAR-Directorate of Rapeseed Mustard Research Sewar, Bharatpur, India; University of Agriculture, PAKISTAN

## Abstract

The ever-increasing industrial activities over the decades have generated high toxic metal such as chromium (Cr) that hampers the crop productivity. This study evaluated the effect of Cr on two chickpea (*Cicer arietinum* L.) varieties, Pusa 2085 and Pusa Green 112, in hydroponic and pot-grown conditions. First, growth parameters (seed germination, seedling growth, and biomass production) and physio-biochemical parameters (oxidative stress and the content of antioxidants and proline) were measured to evaluate the performance of both varieties grown hydroponically for 21 days at concentrations of 0, 30, 60, 90 and 120 μM Cr in the form of potassium dichromate (K_2_Cr_2_O_7_). In both varieties, significantly deleterious effects on germination and seedling growth parameters were observed at 90 and 120 μM, while growth was stimulated at 30 μM Cr. Significant increases in malondialdehyde and hydrogen peroxide content and electrolyte leakage demonstrated enhanced oxidative injury to seedlings caused by higher concentrations of Cr. Further, increasing concentrations of Cr positively correlated with increased proline content, superoxide dismutase activity, and peroxide content in leaves. There was also an increase in peroxisomal ascorbate peroxidase and catalase in the leaves of both varieties at lower Cr concentrations, whereas a steep decline was recorded at higher Cr concentrations. In the pot experiments conducted over two consecutive years, growth, yield, yield attributes, grain protein, and Cr uptake and accumulation were measured at different Cr concentrations. Pusa Green 112 showed a significant reduction in plant growth, chlorophyll content, grain protein, pod number, and grain yield per plant when compared with Pusa 2085. Overall, our results indicate that Pusa 2085 has a higher Cr tolerance than Pusa Green 112. Therefore, Pusa 2085 could be used to further elucidate the mechanisms of Cr tolerance in plants and in breeding programmes to produce Cr-resistant varieties.

## Introduction

Heavy metal (HM) pollution caused by rapid industrialisation and poor management of industrial effluents is among the leading global environmental problems, resulting in the increased HM pollution of agricultural land. HMs usually enter the food chain through plant uptake and accumulation and are passed on to end consumers, and in turn, could lead to numerous health issues [1]. Among HMs, chromium (Cr) causes significant contamination of soil, sediment, and groundwater globally [2]. It enters the soil environment primarily from natural sources such as chromite and volcanoes but also through anthropogenic activities, such as tanning and other industries that produce Cr-containing effluents, mining, electrical equipment waste, and atmospheric deposition [3–6]. In India, cities like Kanpur, Ranipet, Vadodara, and Talcher are heavily polluted by Cr [7]. Recently, the water bodies and catchment area of the Hindon River (a tributary of Yamuna River, India) have been identified as Cr contaminated zones. Polluted water from industries in Ghaziabad city drains into the Hindon River without adequate pre-treatment. This contaminated water from the Hindon River is then utilised for irrigation in the nearby agricultural farmland, thereby affecting the quality of cereals and vegetables grown under such conditions.

Furthermore, Cr deposition in agricultural soils is a global concern as it is non-biodegradable and adversely affects plant growth by decreasing photosynthetic pigments, which results in decreased productivity [8–11]. Cr is classified as a non-essential metal as it does not have any major biological functions [12]. Dotaniya *et al*. [13] reported that increased accumulation of Cr in plants reduces germination as well as the growth rates of roots and shoots, ultimately affecting the total root-shoot biomass and yield. Gill et al. [14, 15] observed a similar trend, with reduced seedling growth and biomass production in *Brassica napus* cultivars following exposure to Cr concentrations in the 0–400 μM range. Many reports have also described Cr effects on plant growth, biomass, and chlorophyll (Chl) content (Chl *a*, *b*, and total), as well as its toxicity to enzyme activity [16, 17]. In addition, excess Cr causes oxidative stress in various plant tissues by increasing the production of reactive oxygen species (ROS), hydrogen peroxide (H_2_O_2_), and malondialdehyde (MDA), and by elevating electrolyte leakage (EL) [18, 19]. Cr-induced oxidative stress causes severe phytotoxic effects in plants by generating ROS at the cellular level [20]. The increased generation of ROS can cause protein and lipid oxidation, nucleic acid impairment, and enzyme inhibition, leading to the damage of cellular components and eventual cell death [21]. In addition, the impacts of Cr toxicity on protein production and Chl concentrations have been reported in *Triticum aestivum* L. and *Pisum sativum* L. [22, 23].

Furthermore, high concentrations of Cr induce the activities of various antioxidant enzymes, namely superoxide dismutase (SOD), catalase (CAT), and ascorbate peroxidases (APX), in plant tissues. Increased SOD activity is considered as the primary step in ROS reduction at the cellular level, while APX and CAT play key roles in limiting the production of H_2_O_2_ [24]. The effect of Cr toxicity on different cultivars of *Brassica napus* under hydroponic condition showed elevation of SOD, POD, APX and CAT activities under the higher concentration of Cr when compared to control [15, 25]. Similar observations were reported in the seedling stage of *Brassica juncea* and *Vigna radiata* where elevated Cr levels were found to enhance the antioxidant activities [2]. These observations suggest that an increased antioxidant enzymes activity acts as a tolerance mechanism to enable the plant to cope with the stress caused by excessive HMs accumulation. Further, exposed plants accumulate Cr predominantly in root tissues, before its translocation to shoots. Previous studies have reported that some plants use roots as the primary storage organ, while others exhibit high heavy metal toxicity tolerance in shoots [26, 27]. Studies on various crops have suggested that knowledge on plant organ Cr tolerance and accumulation should be utilised to identify the edible portions, which would be those accumulating the least HMs [28–31]. In addition to an increase in antioxidant activities, higher proline accumulation was observed in *Vigna unguiculata* seedlings under Cr stress [32].

Chickpea (*Cicer arietinum* L.), also known as Bengal gram, is one of India’s most important pulse crops, with an area of 10.2 M ha under cultivation and a productivity of 11.2 million tonnes and accounting for approximately 44.5% (2017–18) of the national pulse crop production (https://eands.dacnet.nic.in/APY96To06.htm). It is a rich source of protein in the human diet and in animal feed, and maintains soil fertility in wheat-based crop rotation systems in the dry rain-fed areas of the Indian sub-continent, West Asia, and North Africa [33]. Previous studies reported that high levels of Cr and cadmium have negative impacts on plant growth, Chl contents, nitrogen contents in root and shoot tissues, seed production, grain protein contents, and uptake of metals in chickpea [34, 35]. Cr toxicity reduces the availability of essential nutrients, such as sodium, iron, manganese, copper, zinc, and calcium, and negatively affects metabolic processes in the plant, resulting in significant reductions in plant growth and yield [36]. The toxicity and tolerance of Cr were found to vary within crop species and growth development stages. However, there are limited reports on the effects of Cr toxicity in chickpea. Therefore, the present study investigated the effects of different concentrations of Cr on i) morpho-physiological and biochemical changes in two varieties of chickpea grown under hydroponic conditions and on ii) yield and other yield components in the two varieties grown in pots.

## Material and methods

### Plant materials

**Fig 1:**
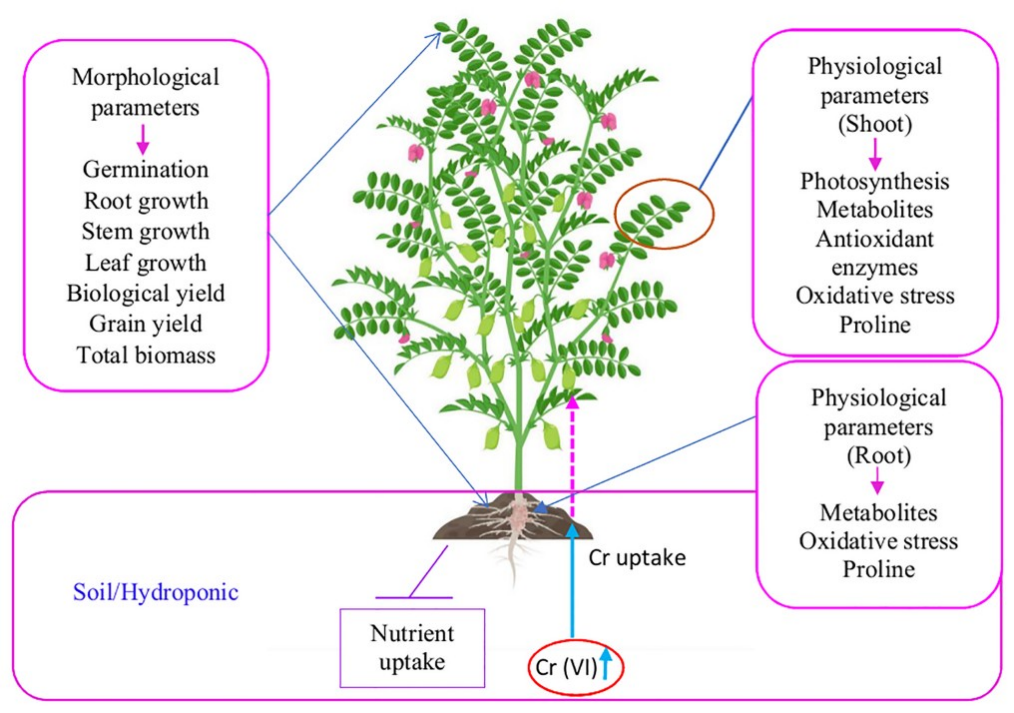
A schematic outlining of mechanism of chromium (Cr) toxicity in soil and the potential deleterious effects of Cr pollution on morphological and physiological aspects of chickpea plants.

Seeds of two chickpea varieties, Pusa 2085 and Pusa Green 112, were obtained from the seed section of the Division of Genetics, ICAR-Indian Agricultural Research Institute, New Delhi, India. Seeds of the same size were selected for the present study. A schematic representation of all the morpho-physiological parameters assessed in both the varieties under Cr Stress is presented in Fig 1.

### Seed germination

Healthy seeds of the two chickpea varieties were sterilised with mercuric chloride (0.2%) solution for 10 min and washed using distilled water. After sterilisation and soaking for 24 h, the seeds were arranged on paper in Petri dishes to evaluate their germination. Using potassium dichromate (K_2_Cr_2_O_7_) as the Cr source, four treatments (30, 60, 90, and 120 μM Cr concentrations) were used to evaluate germination; distilled water was used as the control. Each Petri dish containing 20 seeds was placed in a biochemical oxygen demand incubator (IK 120 E, VELP Scientifica, Italy) set at 22±1 °C. The percentages of seeds that germinated were recorded after 72 h. Germination was considered complete when the emerging radicals were a half of the seed length. Each treatment had three replicates.

### Growth condition and physiological traits under hydroponics

Growth parameters of both varieties were evaluated at the seedling stage under hydroponic conditions. Seven-day-old seedlings of both varieties were used for the experiment conducted under controlled environmental conditions at the National Phytotron Facility, New Delhi, India. The growth chamber was programmed to maintain a day/night air temperature of 30/15 °C (±2 °C), a 10/14 h light/dark cycle, and relative humidity of 60%. The nutrient composition of the medium was as reported by Simon *et al*. [37]. Potassium dichromate was added to the nutrient medium to create the same treatment concentrations as used for the germination test. The experiment was conducted for 21 days, and each treatment was replicated three times, with six plants per replicate. Seedlings’ root and shoot lengths were measured during the experiment. Furthermore, fresh and dry weights of each plant in both varieties were measured.

### Evaluation of chromium toxicity on physio-biochemical parameters

A separate set of experiments were conducted to evaluate the toxic effects of Cr on the physio-biochemical changes of both varieties under hydroponic conditions. The treatment conditions were similar to those established for examining the effects of Cr on growth parameters. The roots and shoots of the treated and control plants of both varieties were harvested to determine the following parameters:

#### Malondialdehyde contents

The MDA contents in tissues (roots and shoots) were measured according to the procedure of Heath and Packer [38]. Fresh root and shoot samples (approximately 0.25 g each) were homogenised using 2 mL of 0.1% trichloroacetic acid and centrifuged at 10,000 ×*g* for 10 min. Subsequently, the supernatant (1mL) was added to 4 mL 0.5% thiobarbituric acid in 20% trichloroethanoic acid, incubated in hot water (95 °C) for 30 min. The reaction was terminated on ice and finally centrifuged at 10,000 ×*g* for 30 min. The absorbance of the supernatant was recorded at 532 and 600 nm, and the MDA contents were deduced by subtracting the nonspecific absorption at 600 nm from the absorption at 532 nm, using an absorbance coefficient of extinction of 155 mM^−1^ cm^−1^.

#### H_2_O_2_ content

The H_2_O_2_ content of the root and shoot samples was measured by homogenising the samples with 10 mL of 50 mM phosphate buffer (pH 7.1). These samples were further centrifuged for 20 min, followed by the addition of 20% sulfuric acid. The samples were centrifuged again for 10 min. The H_2_O_2_ contents were measured by determining the absorbance at 410 nm [39].

#### Detection of electrolyte leakage

Estimation of electrolyte leakage was according to the protocol of Valentovic *et al*. [40]. Briefly, 21-day-old treated and control root and shoot samples were cut into small portions (5 mm) and transferred to test tubes filled with 10 mL deionised water. These test tubes were then incubated in a water bath at 30 °C for 3 h to record the initial electrical conductivity (EC1) and then incubated at 95°C for 1.5 h to record the final electrical conductivity (EC2). After equilibrium at 30°C, EL was estimated using the following equation:

$$\text{EL}=\frac{\text{EL}1}{\text{EL}2}\;\times 100$$

#### Detection of proline

Proline content was estimated according to the method of Bates *et al*. [41]. Fresh root and shoot tissue samples (0.5 g) were collected and homogenised in 3% aqueous sulphosalicylic acid filtered through Whatman filter paper (Praxor, Tamil Nadu, India). Then, 2 mL of glacial acetic acid and 2 mL of ninhydrin were added to the filtered extract (2mL) and boiled in a water bath at 100 °C for 1 h before extraction with 4 mL of toluene. To evaluate the proline content, the absorbance of the chromophore phase was measured at 520 nm using a 1800 UV Spectrometer (Shimadzu Corp. Kyoto, Japan). The quantity of proline was expressed as μM g^−1^ fresh weight (FW).

#### Estimation of antioxidants

Fresh leaf samples were collected from the topmost part of the plant after treatment for 21 days and used for the determination of enzymatic antioxidants. The activity of the antioxidant enzymes, namely SOD, POD, CAT, and APX was determined spectrophotometrically (1800 UV Spectrometer, Shimadzu). Leaf samples (1.0 g) were thoroughly crushed using pre-cooled mortar and pestle and then homogenised in 10 mL of 0.05 M phosphate buffer (pH 7.8), filtered using muslin cloth and centrifuged at 10,000 ×*g* for 15 min at 4 °C. Thereafter, the enzyme extract was used to evaluate SOD and POD activity using the method reported by Zhang [42].

CAT activity was measured according to Aebi [43]. Assay samples (3 mL) consisted of enzyme extract (100 μL), 300 mM H_2_O_2_ (100 μL), and 50 mM phosphate buffer (2.8 mL, pH 7.0) with 2 mM Ca. CAT activity was then determined by measuring the decrease in absorbance at 240 nm as a consequence of H_2_O_2_. APX activity was measured according to the procedures of Nakano and Asada [44]. APX activity was estimated by monitoring the change in the absorbance at 290 nm.

### Evaluation of growth- and yield-related attributes of both varieties in pot experiments

The pot experiments were conducted at the Department of Botany, Meerut College, Meerut, Uttar Pradesh, India, for two consecutive years (2018–19 and 2019–20) to ensure the reproducibility of the results. Treatment conditions were similar to those of the hydroponic experiments but under rainfed conditions. Seeds of both varieties were sown and grown in PVC pots (height 30 cm; area 24 cm^2^) filled with 5 kg of sandy loam soil. The initial physicochemical properties of the soil are listed in S1 Table. Seven pots were used for each treatment (control and the four different Cr concentrations) in each variety. The experiment was set up based on a completely randomised design with three replicates for each treatment for two consecutive years. The leaves of both varieties were sampled after 95 days of treatment to determine the Chl and nitrogen content of tissues (roots and shoots). The remaining plants were allowed to grow to maturity and for the examination of growth and yield components, as well as Cr accumulation in different organs.

#### Estimation of chlorophyll content

After 95 days of treatment, 0.5 g samples of healthy leaves from the upper portions of plants were used to estimate Chl *a* and Chl *b* contents. The Chl was extracted in 85% v/v acetone solution (Sigma Aldrich, St. Louis, MI, USA) and kept in the dark at 4 °C until the green colour disappeared. The extracts were then centrifuged at 3000×*g* for 10 min at 4 °C, and the absorbance of the supernatant was recorded at 663 nm and 645 nm for Chl *a* and Chl *b*, respectively, using a spectrophotometer (1800 UV Spectrometer, Shimadzu) [45]. Chl content was determined according to Arnon [46] and expressed as mg g^−1^ FW. The total Chl content was calculated as follows:

$$\text{Total}\;\text{Chl}\;(\text{mg}\;\text{g}-1\;\text{FW})=20.2\;(\text{OD}\;\text{at}\;663\;\text{nm})+8.02\;(\text{OD}\;\text{at}\;645\;\text{nm})\times \frac{\text{V}}{1000\times \text{W}},$$

where V is the final volume, OD is the optical density, and W is the fresh weight of samples.

#### Estimation of nitrogen content

The root and shoot nitrogen contents were estimated 95 days after the onset of treatments using the micro-Kjeldahl method according to Iswaran & Marwah [47]. First, each sample (50 mL) was placed in a Kjeldahl flask containing 5 mL of water and 15 mL of nitrogen/100 mL sulfuric acid, and shaken vigorously. Drop-wise addition of 0.1N KMNO_4_was carried out until a pink colour was observed. The catalyst mixture (3 g K^-^SO_4_, 0.3 g FeSO_4_.5H_2_O, and 0.15 g CuSO_4_.5H_2_O) was then added, and the reaction was performed on low flame for 30 min until the mixture developed yellowish green colour.

#### Estimation of grain protein (GP)

Both varieties were harvested 140 days after sowing (DAS), and the total protein content of the seeds was measured using Lowry’s method [48]. To measure GP, 500 mg of seeds were saturated in phosphate buffer (pH 7.4), ground, and placed in 5−10 mL phosphate buffer (pH 7.4). Proteins were extracted with 0.1 M sodium hydroxide and treated with Folin phenol reagent, which turned blue. A calibration curve was generated using bovine serum albumin as the standard curve, and GP was expressed as mg g^-1^ seed FW.

#### Growth- and yield-related attributes

At 140 DAS, seven plants were selected from each treatment in the two varieties and their growth (plant height, number of primary and secondary branches, fresh and dry mass) and yield attributes were evaluated. Immediately after harvesting, the FW of plant samples from the same treatments were measured and the samples were placed in an oven at 75–80 °C for 12 h. Grain yield was recorded on a 100-grain weight basis (i.e. 100 grains were randomly selected and weighed).

#### Chromium accumulation

To measure Cr content, plant tissues (roots, stems, leaves and grains) were transferred to 100-mL flasks, to which 15 mL concentrated HNO_3_ was added. The mixtures were heated on a hot plate and the temperature was slowly and steadily increased to 275 °C until dense yellow fumes were formed. When the formation of dense yellow fumes decreased, H_2_O_2_ was added to the mixture and cooled. Distilled water was then added up to a final volume of 25 mL. Cr concentrations were determined by atomic absorption spectrometry using a ZEEnit 700P spectrophotometer (Analytik Jena AG, Jena, Germany). The concentrations and accumulation of Cr in each part of the tissues were measured according to Jabeen et al. [30].

### Statistical analyses

Different Cr concentrations had varying effects on the morpho-physiological, growth, and yield parameters of both the chickpea varieties. The results obtained were compared using one-way analysis of variance and multiple comparison procedures available in the Statistical Analysis System (SAS, version 9.4; SAS Institute, Cary, NC, USA). Pearson’s correlation coefficients were used to evaluate the associations between different traits at different stages of growth. Graphs were illustrated using Statgraphics 18 (https://www.statgraphics.com) and R software (https://www.r-project.org/). In all procedures, a p < 0.05 was considered significant.

## Results

In the present study, chickpea plants were exposed to different concentrations of Cr. Different morpho-physiological and biochemical attributes, including changes in plant growth, Chl content, oxidative stress, antioxidant enzymes and metabolites activities were deduced. The total Cr uptake and its accumulation by plants were also examined. The results are presented below.

### Seed germination under the different Cr concentrations

**Fig 2:**
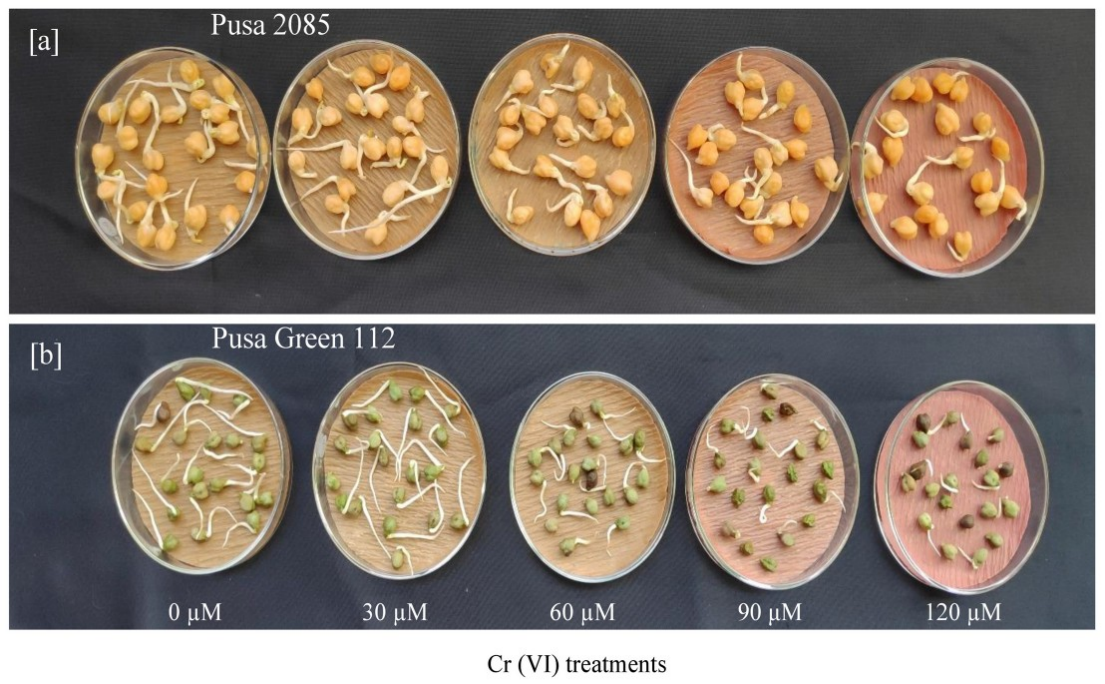
Germination performance of seeds in two chickpea varieties under different chromium (Cr) treatments. (a) Pusa 2085. (b) Pusa Green 112.

Exposure to Cr caused a significant reduction in the germination percentage. In the 120-μM treatment, seed germination of both varieties was significantly affected (Fig 2). Compared to the control, germination was reduced by 53.13% and 57.80% in Pusa 2085 and Pusa Green 112, respectively. In the 30-μM Cr treatment, there was no significant difference between the control and Pusa 2085 but a significant reduction was noted in Pusa Green 112 (S2 Table). In the 120-μM Cr treatment, the germination index showed a significant decrease of 31.63% and 32.75% for Pusa 2085 and Pusa Green 112 respectively, compared to the control. There was also a significant decrease in the vigour index of both the chickpea varieties, compared to the control (S1 Fig). In response to treatment with the 120 μM Cr, a greater reduction in the germination and vigour indices were observed in Pusa Green 112 than in Pusa 2085, indicating that the latter could tolerate the effects of higher levels of Cr more efficiently.

### Effect of various concentrations of Cr on seedling growth under hydroponic conditions

**Fig 3:**
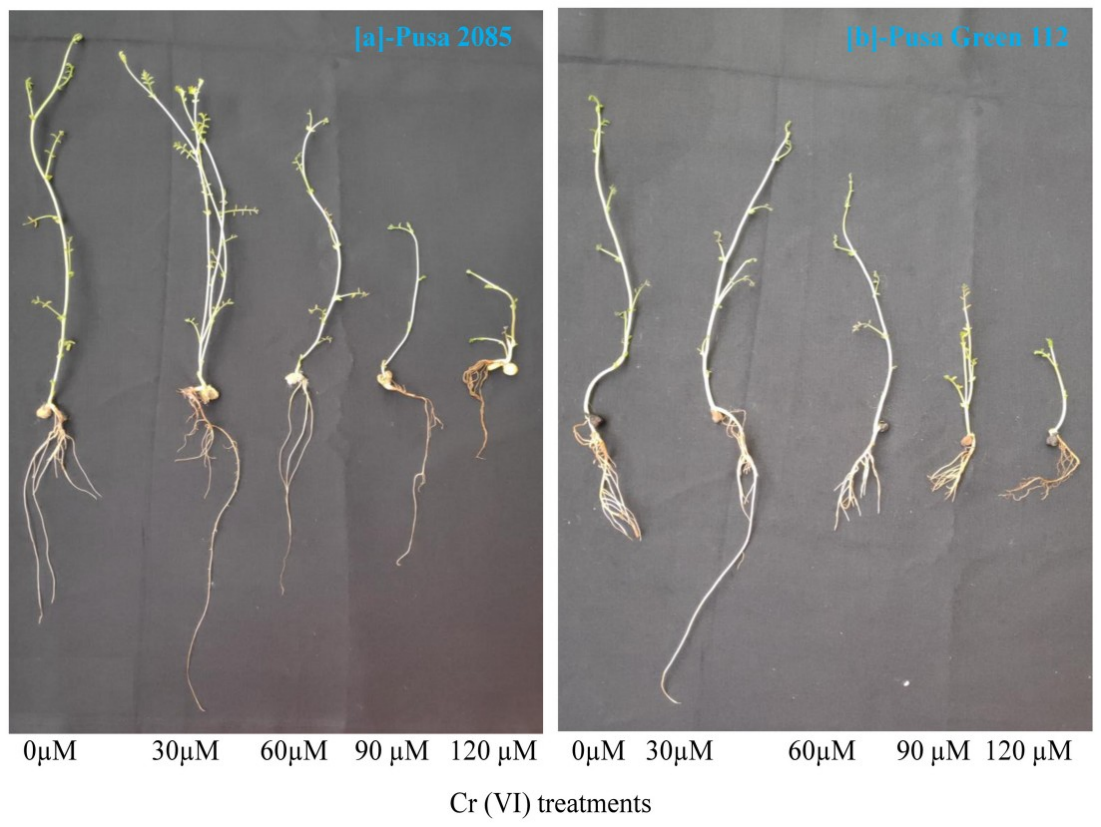
Effects of various chromium (Cr) concentrations on the morpho-physiological traits of two chickpea varieties under hydroponic conditions. (a) Pusa 2085. (b) Pusa Green 112.

**Fig 4:**
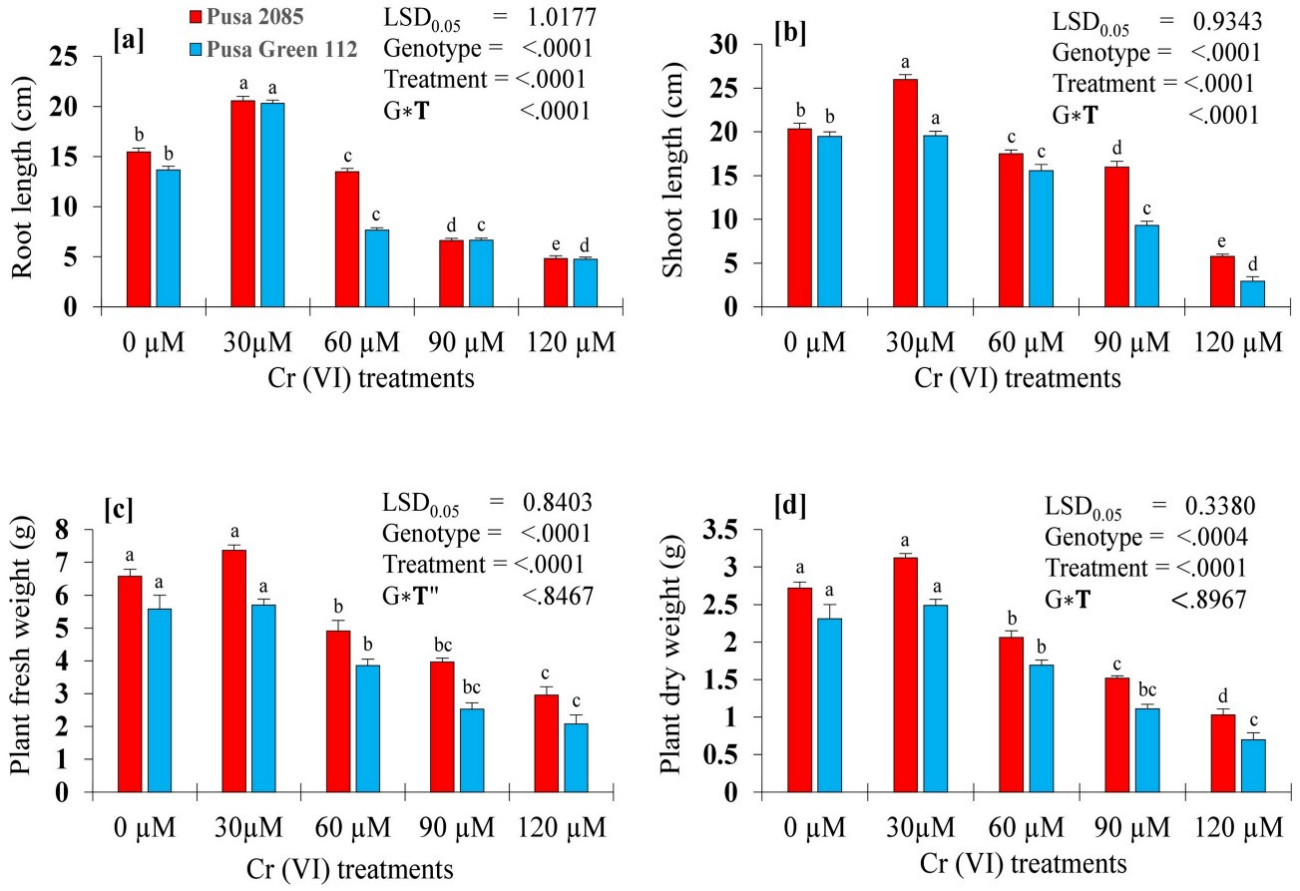
Effects of the various chromium (Cr) concentrations on two chickpea varieties (Pusa 2085 and Pusa Green 112) 21 days after treatment (DAT). (a) root length (cm). (b) shoot length (cm). (c) plant fresh weight (g). (d) plant dry weight (g). Values are means ± standard errors from three independent experiments. Error bars with the same letters are not significantly different at p ≤ 0.05.

In the present study, the toxic effects of different Cr concentrations on seedling growth parameters, including root and shoot lengths, and plant fresh and dry weights, in two chickpea varieties, were investigated. In both varieties, seedling growth was less affected at lower Cr concentrations than at higher Cr concentrations. However, all seedling growth characteristics were affected by Cr treatments when compared with the control seedlings (Fig 3). In response to the 120-μM Cr treatment, reductions of 68.71% and 65.25% in root length and 71.63% and 84.86% in shoot length were observed for Pusa Green 112 and Pusa 280, respectively (Fig 4a and 4b), indicating that shoots were more adversely affected than roots. Further, increasing Cr concentration from 60 to 120 μM reduced the growth-related parameters of both chickpea varieties. Further, in response to the 120-μM Cr treatment, the FWs of plants were reduced by 55.02% and 62.72% in the Pusa 2085 and Pusa Green 112 varieties, respectively, in comparison with the control plants. The plant dry weight followed the same trend and decreased by 62.13% and 69.70% respectively, compared to the control (Fig 4c and 4d). In both varieties, biomass production showed the most significant decrease in response to the 120 μM treatment. As the Cr uptake increased, the biomass production of the plant decreased. However, the biomass production of both varieties also increased in response to the lowest Cr concentration (30 μM; Fig 4c and 4d). Further, the reduction in root length, shoot length, and biomass production of plants was lower in Pusa 2085 than in Pusa Green 112 (Fig 4a–4d). Collectively, Pusa 2085 showed higher tolerance to Cr than Pusa Green 112.

### Changes in oxidative stress and electrolyte leakage

**Fig 5:**
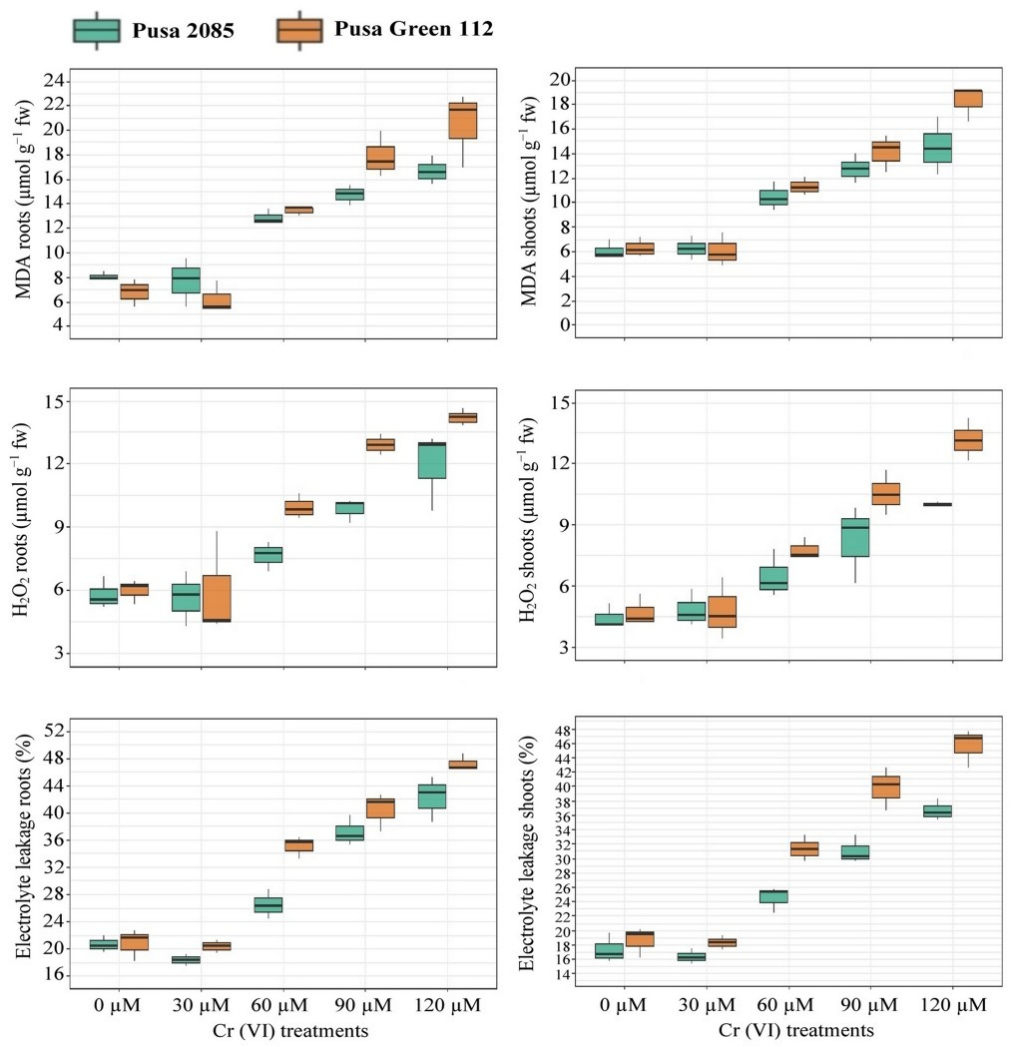
Effects of various chromium (Cr) concentrations on two chickpea varieties (Pusa 2085 and Pusa Green 112). (a) Malondialdehyde (MDA), roots. (b) MDA, shoots. (c) H_2_O_2_, roots. (d) Hydrogen peroxide (H_2_O_2_), shoots. (e) electrolyte leakage (EL), roots. (f) electrolyte leakage, shoots.

Root and leaf samples were examined to determine changes in MDA and H_2_O_2_ production, and EL (Fig 5). It was observed that in response to 120 μM Cr, the production of MDA in the roots and shoots of Pusa 2085 increased by 51.71% and 58.20%, respectively, compared with the control. A similar trend was observed for Pusa Green 112 under 120 μM Cr but Pusa 2085 showed lower accumulation of MDA in roots and shoots in all treatments as compared to Pusa Green 112 (Fig 5a and 5b). Further, in response to the highest Cr treatment, the H_2_O_2_ content in the roots and shoots of Pusa 2085 increased by 51.34% and 55.44%, respectively, whereas in Pusa Green 112 it increased by 60.38% and 63.98%, respectively, compared to the controls (Fig 5c and 5d). In both varieties, EL increased in response to Cr. In the 120-μM Cr treatment, the EL value increased by 42.21% and 36.58% in the roots and shoots, of Pusa 2085, respectively, and by 47.17% and 45.58% in that of Pusa Green 112 (Fig 5e and 5f). Overall, EL was lower in Pusa 2085 than in Pusa Green 112. The results also indicated that exposure to Cr up to a concentration of 120 μM resulted in a significant increase in MDA, H_2_O_2_, and EL in roots and shoots of both Pusa 2085 and Pusa Green 112.

### Proline content

**Fig 6:**
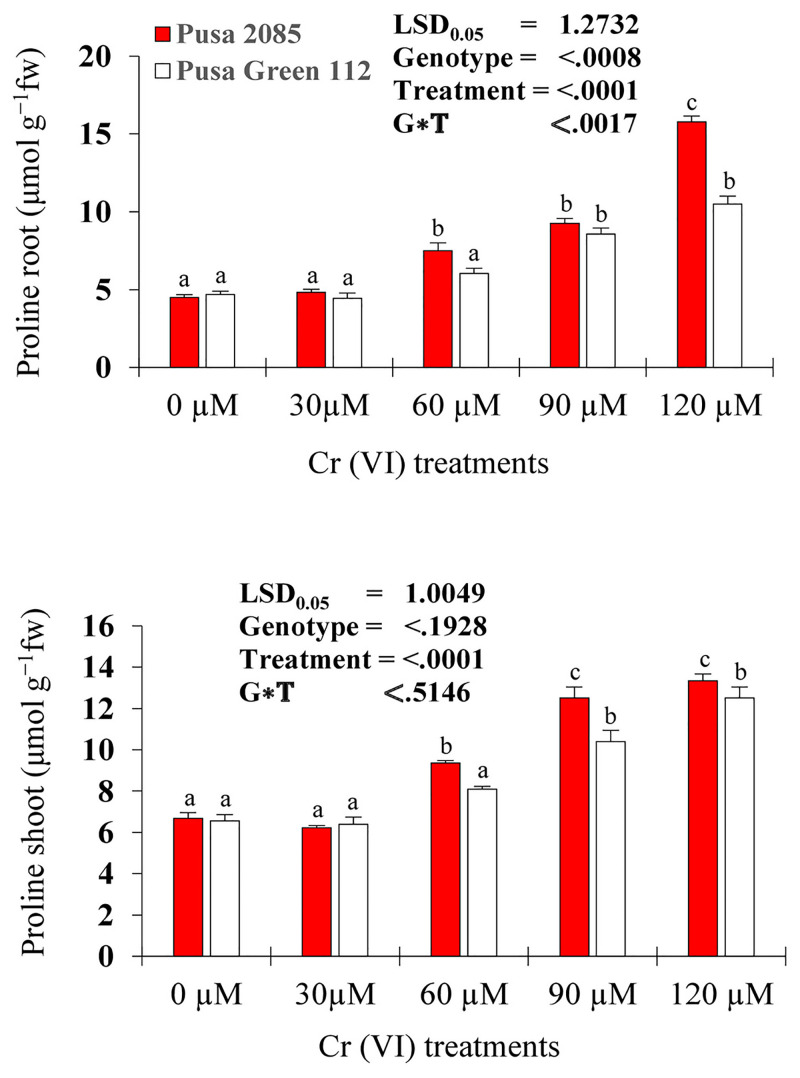
Effects of the various chromium (Cr) concentrations on the proline contents in roots and shoots of two chickpea varieties (Pusa 2085 and Pusa Green 112) 21 days after treatment (DAT). Values are means ± standard errors from three independent experiments. Error bars with the same letters are not significantly different at p ≤ 0.05.

The estimation of proline content showed that as Cr concentration increased, proline content also increased in the roots and shoots of both varieties (Fig 6). In response to the 120-μM Cr treatment, the proline content in the roots and shoots of Pusa 2085 increased by 71.56% and 49.93%, respectively, compared to the control. Following a similar trend, Pusa Green 112 showed an increase of 55.24% and 47.56% in roots and shoots, respectively. Overall, Pusa 2085 exhibited a higher proline content than Pusa Green 112 (Fig 6).

### Performance of the antioxidant system

**Fig 7:**
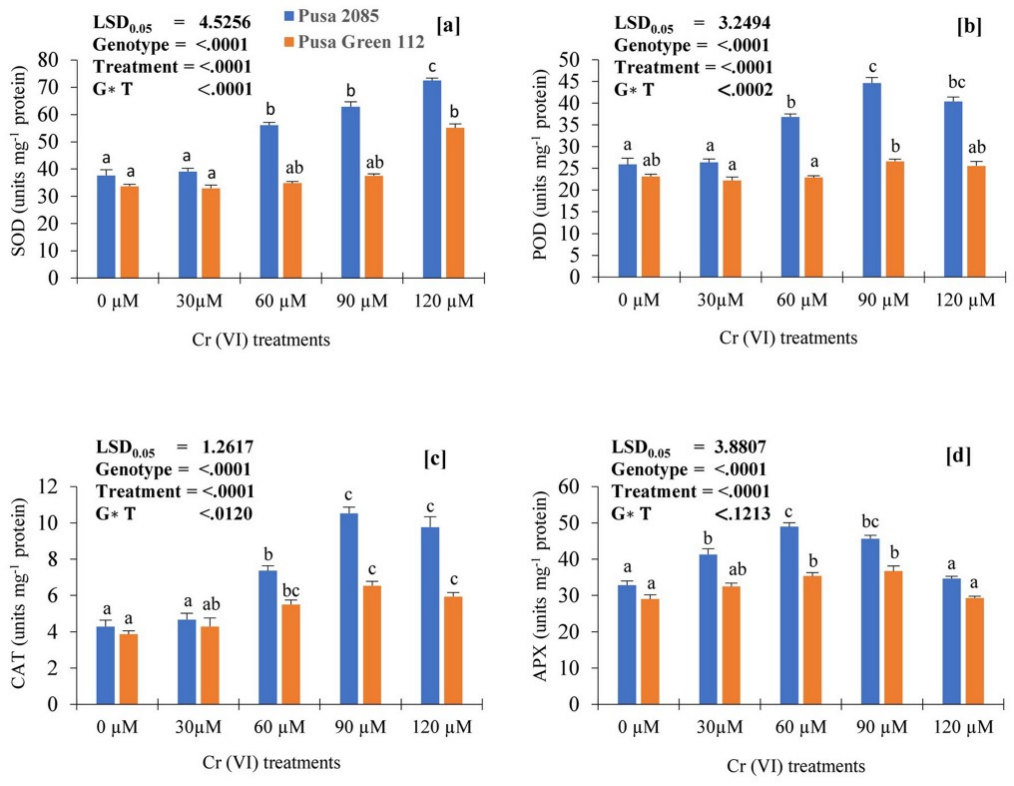
Effects of the various chromium (Cr) concentrations on the activities (units mg^-1^ protein) of antioxidative enzymes: Superoxide dismutase (SOD), Peroxidase (POD), Catalase (CAT), and Ascorbate Peroxidase (APX) in the leaves of two chickpea varieties (Pusa 2085 and Pusa Green 112) 21 days after treatment (DAT). Values are means ± standard errors from three independent experiments. Error bars with the same letters are not significantly different at p ≤ 0.05.

SOD, CAT, APX, and POD activities in the leaves of both varieties (Fig 7) increased with increasing Cr concentrations. Compared with the control, the SOD and POD activities in Pusa 2085 increased by 48.06% and 35.88%, respectively, whereas in Pusa Green 112, they increased by 63.59% and 9.53%, respectively. Furthermore, in the 120-μM Cr treatment, CAT activity increased by 56.09% and 34.85 in the leaves of Pusa 2085 and Pusa Green 112, respectively (Fig 7c). APX activity increased by 5.28% and 1.12% in the leaves of Pusa 2085 and Pusa Green 112 varieties, respectively, under the 120-μM Cr treatment (Fig 7d). Overall, and in both varieties, APX, CAT, and POD activities increased in response to the lowest Cr concentration (30 μM) but decreased as the Cr concentration increased (Fig 7b–7d). CAT activity in the leaves of Pusa 2085 was significantly higher at high Cr concentrations than in Pusa Green 112. All enzymatic activities were higher in Pusa 2085 than in Pusa Green 112 (Fig 7a–7d) indicating that Pusa 2085 could tolerate oxidative stress more effectively than Pusa Green 112 under the Cr concentrations tested.

### Growth- and yield-related attributes in the pot experiment

**Fig 8:**
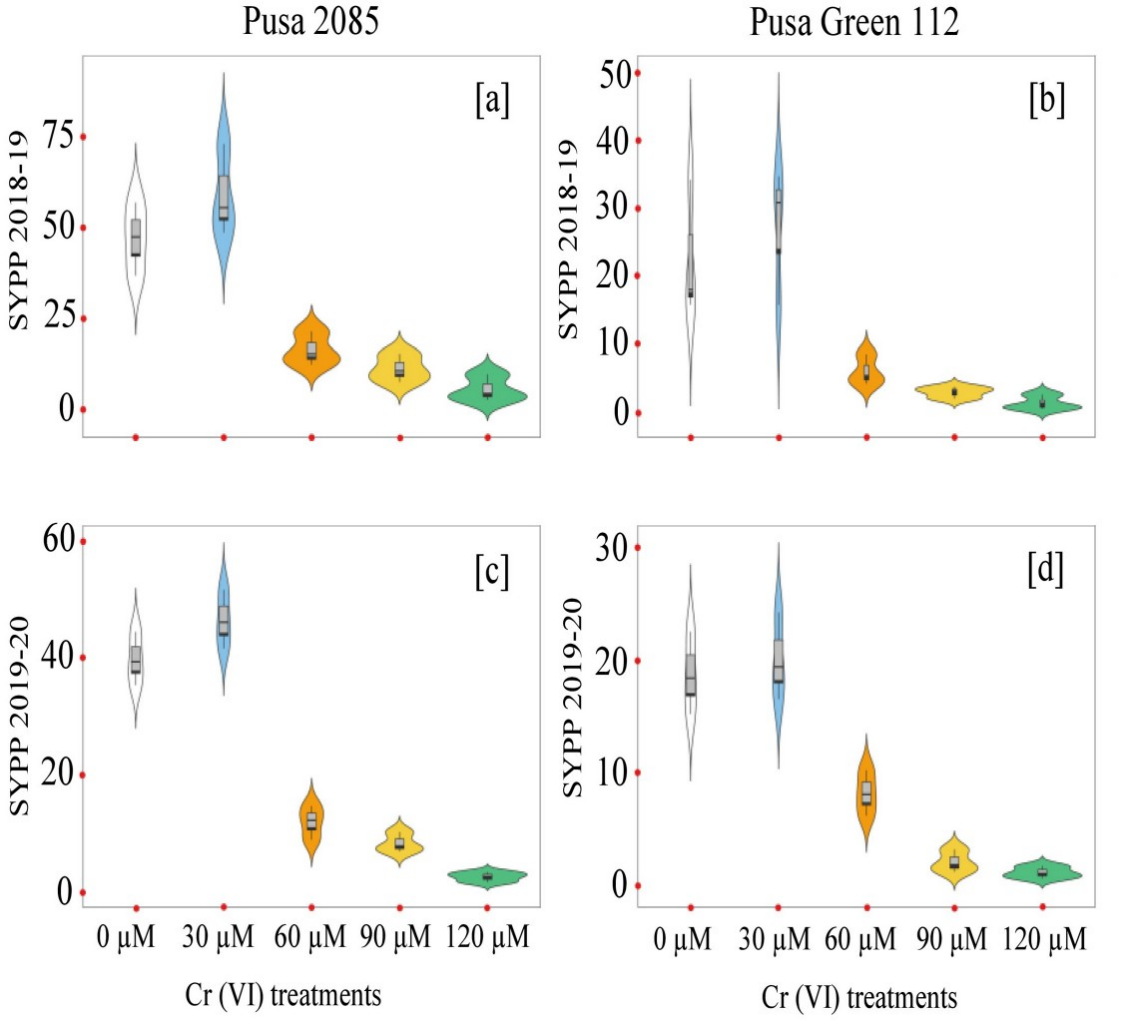


**Fig 9:**
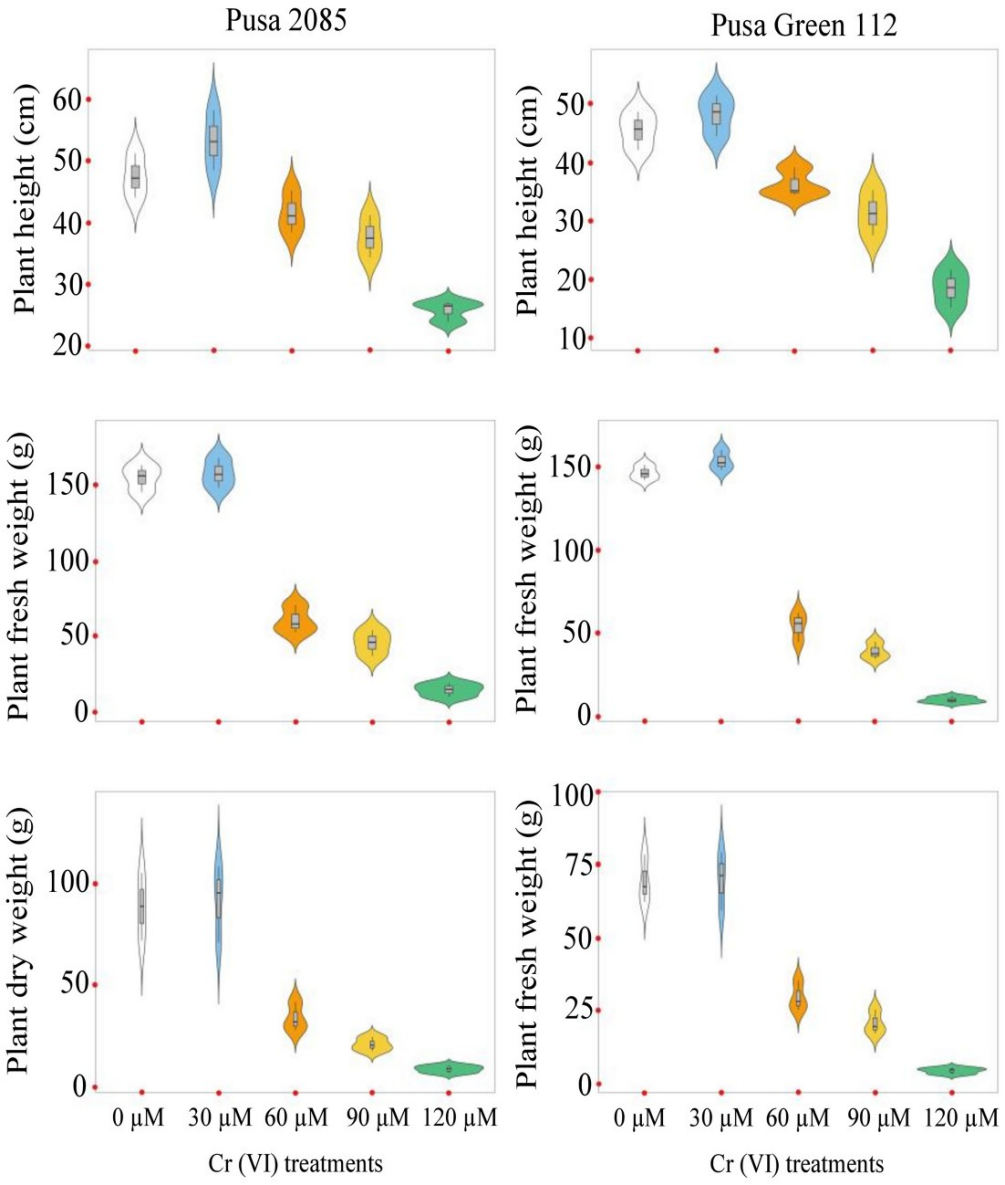
Violin plots showing the distributions of three plant growth parameters examined in two chickpea varieties, Pusa 2085 and Pusa Green 112, grown in pots under different chromium (Cr) concentrations. (a–b) plant height (PH) (cm). (c–d) plant fresh weight (PFW) (g). (e–f) plant dry weight (PDW) (g).

**Fig 10:**
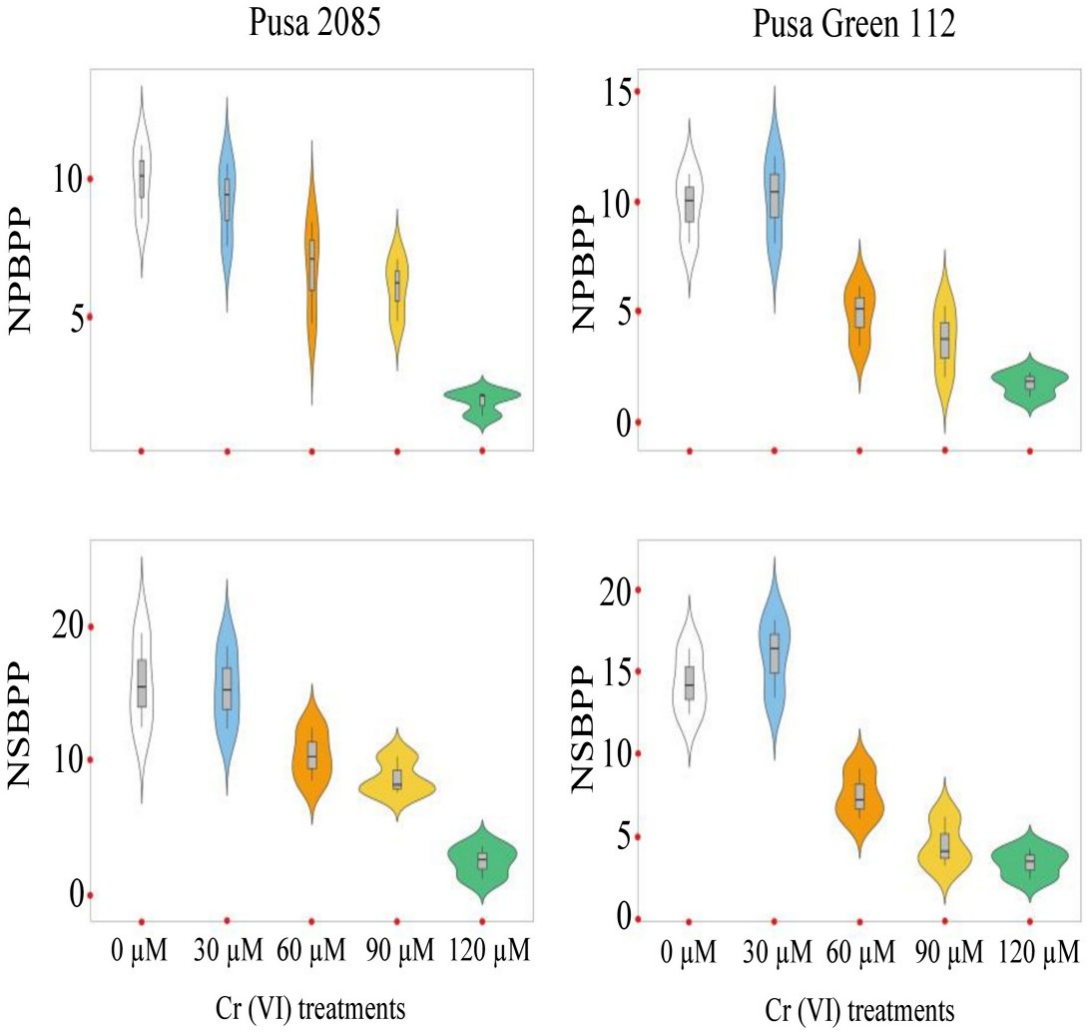
Violin plots showing the distributions of the number of primary branches per plant (NPBPP) and the number of secondary branches per plant (NSBPP) in two chickpea varieties, Pusa 2085 and Pusa Green 112, grown in pots under different chromium (Cr) concentrations.

**Fig 11:**
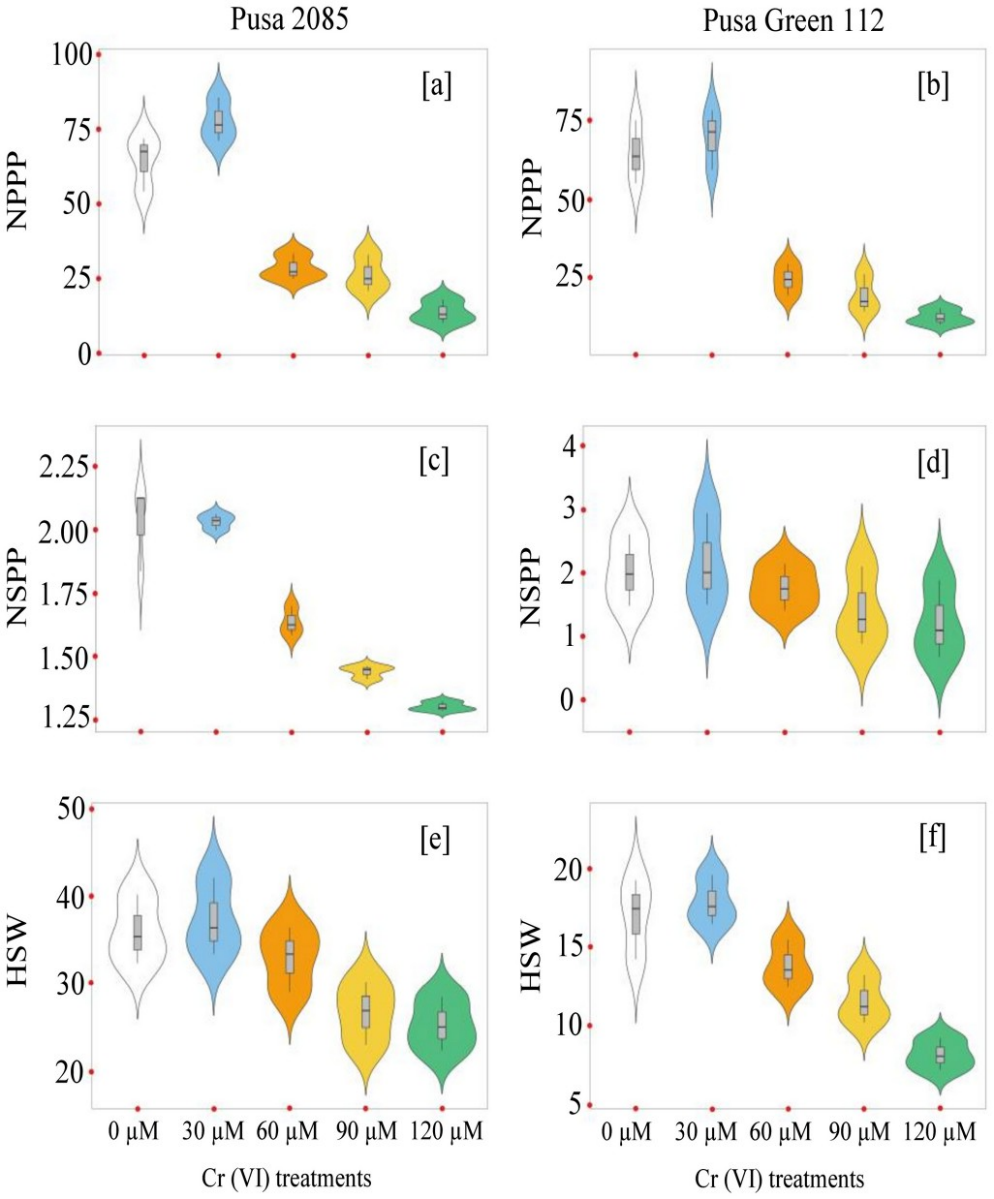
Violin plots showing the distributions of the number of pods per plant (NPPP) and the yield-related traits in two chickpea varieties, Pusa 2085 and Pusa Green 112, grown in pots under different chromium (Cr) concentrations.

**Fig 12:**
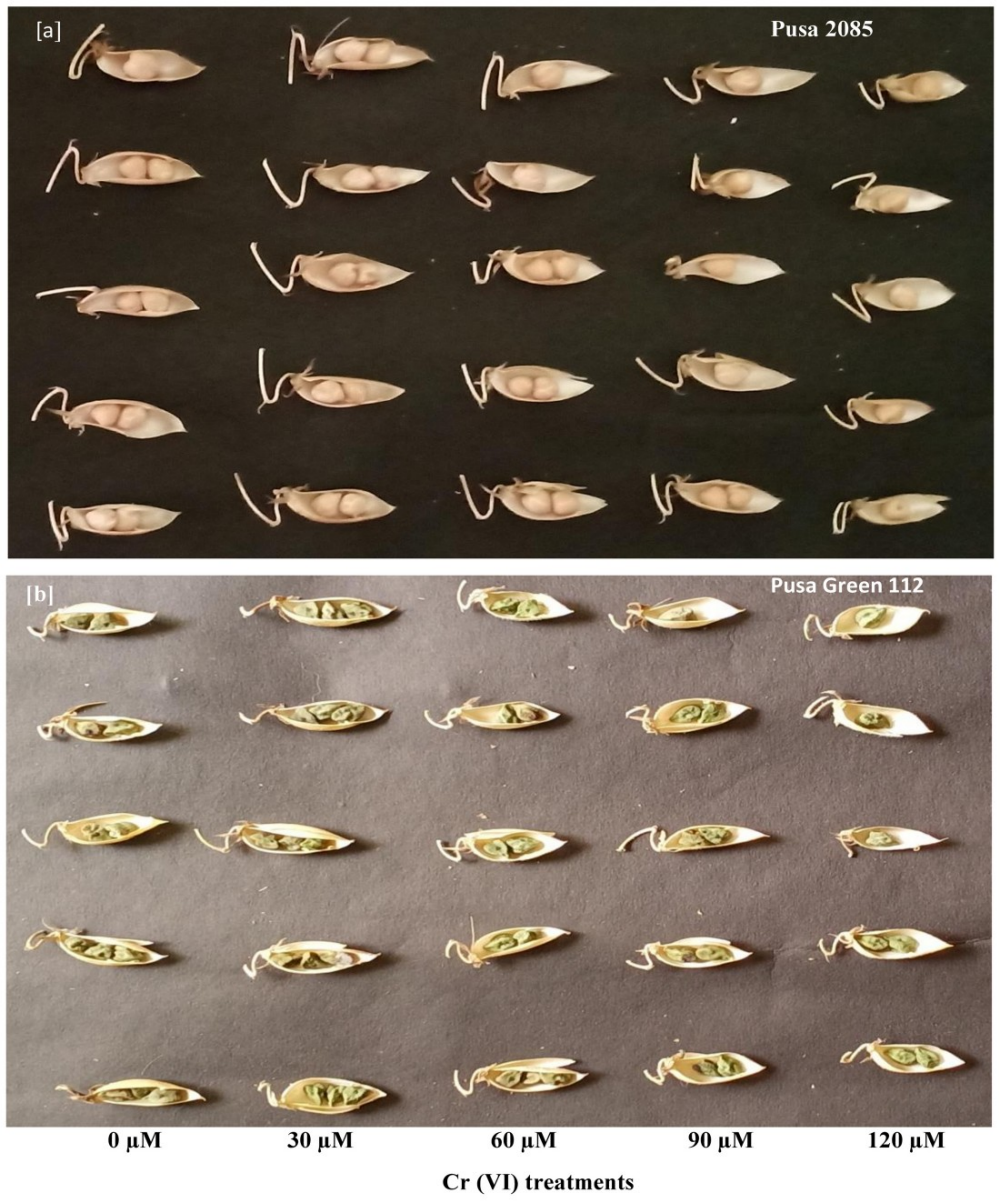
Effects of various chromium (Cr) concentrations on the seeds per pod in two chickpea varieties grown under potted conditions. (a) Pusa 2085. (b) Pusa Green 112.

The effects of Cr toxicity on growth, yield, and yield-related components of both chickpea varieties were investigated. The genotypes responded differently to different Cr treatments, and the reduction in seed yield per plant varied. The values for Pusa 2085 and Pusa Green 112 in the different Cr treatments were: 16.42 and 12.17 g at 60 μM, 11.21 and 8.55 g at 90 μM, and 5.59 and 2.78 g at 120 μM Cr, respectively, in 2018–19 (Fig 8a and 8b). A similar trend was observed during 2019–20 but the reduction in seed yield per plant was higher compared to the previous year (Fig 8c and 8d). The Pusa 2085 genotype showed a lower reduction in pod and seed yield per plant than the Pusa Green 112 genotype. It showed a significantly greater reduction in pod and seed yield per plant compared to the controls. At high Cr concentrations, Pusa 2085 showed significant reductions in plant height (45.48%, p < 0.05), plant fresh biomass (90.34%, p < 0.05), plant dry biomass (89.41%, p < 0.05), number of primary branches per plant (80.70%, p < 0.05), number of secondary branches per plant (84.44%, p < 0.05), number of pods per plant (78.33%, p > 0.05), and number of seeds per pod (Figs 9–12). Similar results were observed in Pusa Green 112. The 100-grain weight was also affected by the Cr treatments (Fig 11e and 11f).

**Fig 13:**
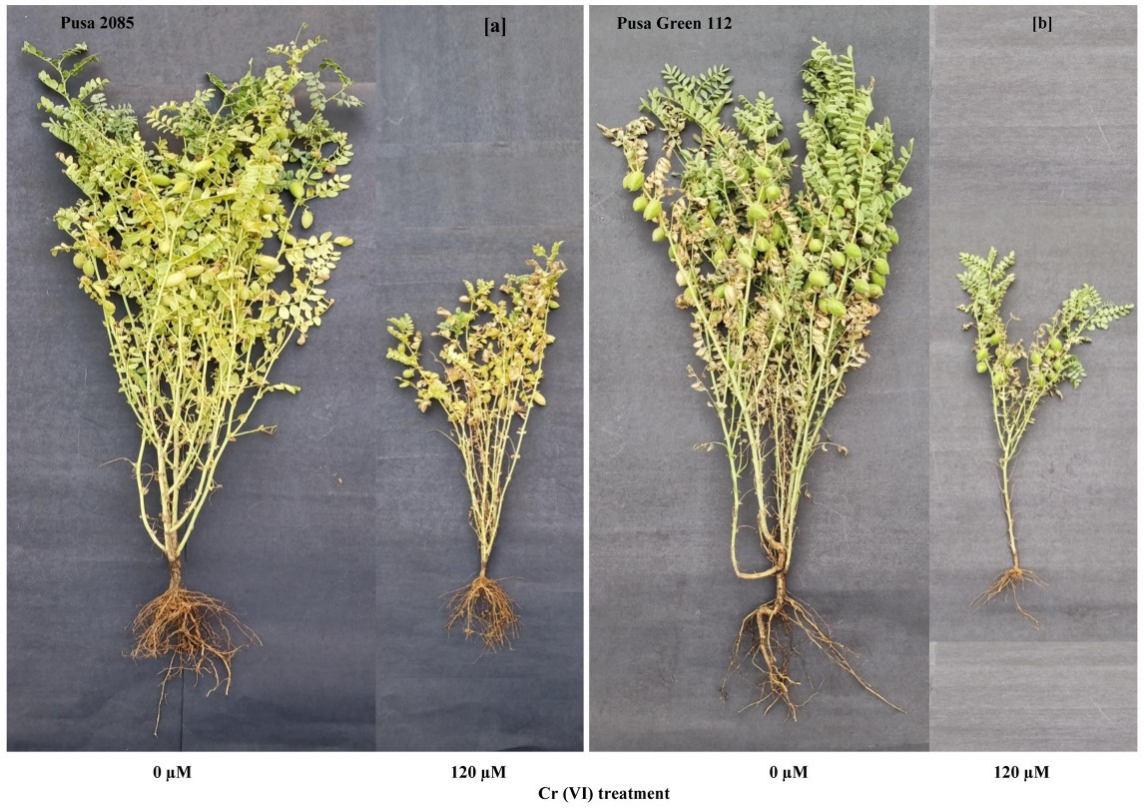
Effects of the chromium (Cr) treatments (control and 120 μM) on plant growth, above-ground biomass, and plant productivity in two chickpea varieties grown in pots. (a) Pusa 2085. (b) Pusa Green 112.

Increased Cr stress decreased the 100-grain weight by 29.57% and 51.68% (p < 0.05) in Pusa 2085 and Pusa Green 112, respectively. In general, the 100-grain weight of Pusa 2085 was higher than that of Pusa Green 112. The results of the current research indicate that high Cr levels affected plant height, number of primary and secondary branches per plant, total biomass, and number of pods per plant. However, the number of primary and secondary branches per plant, plant height, pod and seed yield per plant, and biomass production in either of the two varieties, were not significantly affected at the lower (30 μM) concentration of Cr. High-concentration treatments had significantly higher negative impacts on plant productivity in Pusa Green 112 than in Pusa 2085 (Fig 13).

### Changes in chlorophyll contents

**Fig 14:**
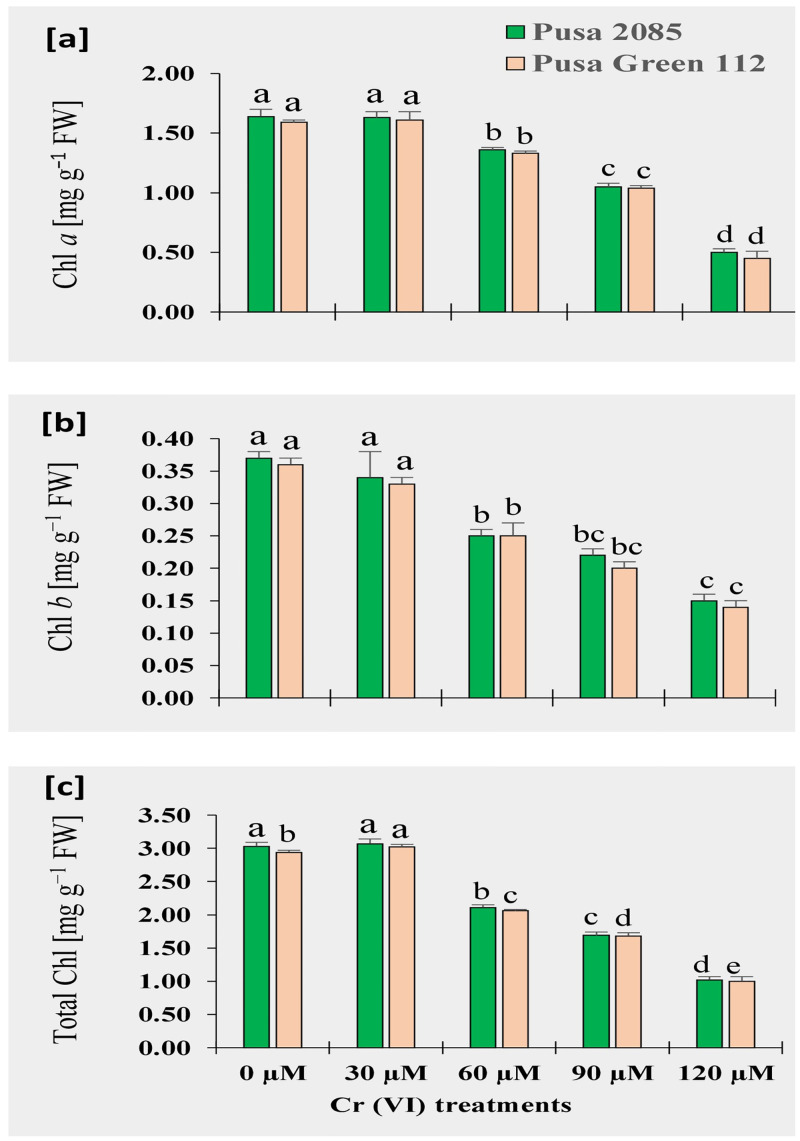
Effects of various chromium (Cr) concentrations on chlorophyll (Chl) contents of leaves in two chickpea varieties (Pusa 2085 and Pusa Green 112), grown in pots, 95 days after treatment (DAT). (a) Chl *a*. (b) Chl *b*. (c) Total Chl. Values are means ± standard errors from three independent experiments. Error bars with the same letters are not significantly different at p ≤ 0.05.

Photosynthetic pigments (Chl *a*, Chl *b*, and total Chl) were significantly affected at 60 to 120 μM Cr concentrations when compared with the controls (Fig 14). Decreases in the contents of all three types of Chl were observed with increasing Cr concentrations. In Pusa 2085, the maximum Chl *a* and *b* (1.64 and 0.37 mg g^−1^ FW, respectively) were recorded in the leaves of control plants, whereas total Chl was highest (1.61 mg g^−1^ FW) at 30 μM Cr (Fig 14a–14c). Similarly, in the leaves of Pusa Green 112, Chl *a* and total Chl contents were increased in the 30 μM Cr concentration; however, the Chl *b* content was highest in control plants (Fig 14). Overall, high Cr concentrations (60 to 120 μM) significantly affected Chl contents, but at the lowest concentration (30 μM) it did not affect the Chl content of either variety. Furthermore, Pusa 2085 showed higher Chl *a*, Chl *b*, and total Chl contents under increasing Cr concentrations than Pusa Green 112 (Fig 14a–14c).

### Nitrogen content

The nitrogen content in the roots and shoots of the both varieties was significantly decreased in pot conditions in the 120-μM Cr treatment. In Pusa 2085, the reductions in the nitrogen content of roots and shoots were 71.03% and 75.60%, respectively, in the 120-μM Cr treatment compared with the control. Similar results were observed for Pusa Green 112. Nitrogen content decreased in the roots and shoots of both the varieties in all treatments but decrease was higher in Pusa Green 112 than in Pusa 2085 (S2 Fig). There was no significant difference in the nitrogen content of roots and shoots in the controls of both varieties. Overall, in Cr- treated seedlings, the nitrogen content of roots and shoots was lower in Pusa Green 112 than in Pusa 2085, and both were significantly lower than in the controls. Furthermore, there were no significant differences between the controls and seedlings of both varieties in the low-concentration Cr treatment with respect to the nitrogen content in roots and shoots. The nitrogen content of chickpea seedlings significantly decreased with increasing Cr concentrations (S2 Fig).

### Changes in grain protein

The effect of Cr on the GP content of chickpea seeds is shown in S3 Fig. The evaluation of GP indicated a significant reduction in the 120-μM Cr treatment compared with the control plants. The 30-μM Cr treatment had a stimulatory but non-significant effect on the GP content of both Pusa 2085 and Pusa Green 112. Compared with the controls, in the 120-μM Cr treatment, there was a decrease in GP by 53.06% and 66.89% in Pusa 2085 and Pusa Green 112, respectively. Although it has been observed that Cr decreased GP concentrations in both Pusa Green 112 and Pusa 2085 in the 60- to 120-μM Cr treatments (S3 Fig), the results also indicated that Pusa 2085 had a higher tolerance to Cr than Pusa Green 112.

### Cr uptake and accumulation in different plant organs

**Fig 15:**
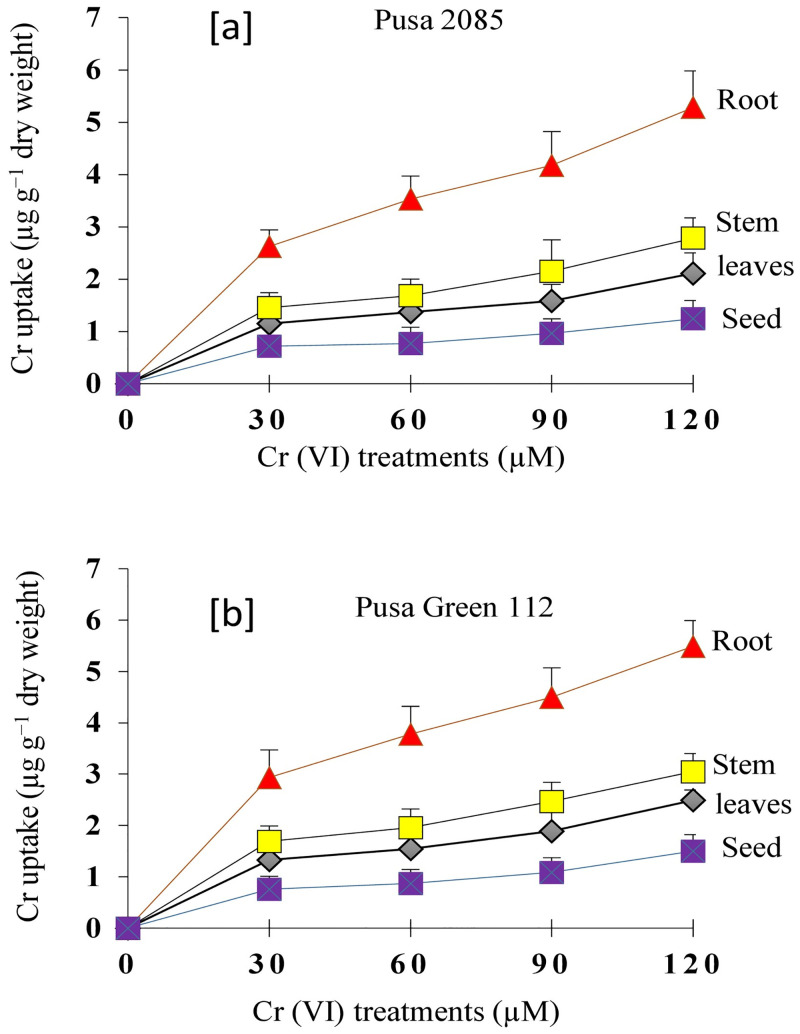
Chromium (Cr) accumulation in roots, stems, leaves, and seeds of Pusa 2085 and Pusa Green 112 chickpea seedlings grown in pots under different Cr concentrations (0, 30, 60, 90 and 120 μM).

The Cr content of leaves, stems, roots, and seeds of both chickpea varieties at 140 DAS was measured. All organs of both varieties showed a significant increase in Cr content with an increase in the Cr concentration of the treatments (Fig 15a and 15b). For Pusa 2085, the Cr content of roots, stems, leaves, and seeds were 4.59, 2.78, 2.11, and 1.24 μg g^−1^ dry weight, respectively, in the 120-μM Cr treatment. Similarly, for Pusa Green 112, increased Cr concentrations caused an increase of Cr content in the same plant organs (Fig 15b). The highest accumulation of Cr was observed in the roots, followed by the stems, leaves, and seeds. In both varieties, the increase in Cr content was higher in the roots and in the 120-μM Cr treatment, suggesting that chickpea’s roots are the principal organ for the accumulation and storage of Cr and that its toxic effect on the roots diminishes plant growth and production (Fig 13).

### Pearson’s correlations

Growth attributes (plant height, fresh and dry weights, and number of primary and secondary branches) and yield attributes exhibited variable Pearson’s correlations within both varieties (Table 1). There was a negative correlation between the Cr content of the plant organs and all growth and yield attributes. The highest and lowest variations were observed in the 120-μM and 30-μM Cr treatments. Negative correlations were observed between the Cr content and both plant growth and Chl content of leaves; nitrogen contents of roots and shoots were positively correlated with both plant growth and Chl content (Table 1). Significant correlations were observed between Cr concentrations and all other measured parameters associated with plant growth and performance. Overall, the results indicated that Pusa 2085 could be used in the investigation of the mechanisms of Cr tolerance and in breeding programmes to generate Cr-resistant varieties.

**Table 1:** Pearson correlation coefficients between different morpho-physiological traits and growth and yield-related attributes and Cr uptake from among parts of (i) Pusa 2085 and (ii) Pusa green 112 genotype under pots conditions.

**(i)**																			
	PH	PFW	PDW	NPB	NSB	Chl a	Chl b	Total Chl	NR	NS	Cr-Root	Cr-Leaves	Cr-stem	Cr-Seed	NPPP	NSPP	HSW	SY18-19	SY19-20
PH	1.000																		
PFW	0.885**	1.000																	
PDW	0.869**	0.968**	1.000																
NPB	0.883**	0.894**	0.844**	1.000															
NSB	0.935**	0.868**	0.827**	0.841**	1.000														
Chl a	0.931**	0.889**	0.843**	0.918**	0.931**	1.000													
Chl b	0.856**	0.919**	0.948**	0.814**	0.823**	0.870**	1.000												
Total Chl	0.926**	0.972**	0.934**	0.908**	0.917**	0.964**	0.929**	1.000											
NR	0.834**	0.957**	0.918**	0.843**	0.853**	0.899**	0.915**	0.965**	1.000										
NS	0.839**	0.923**	0.901**	0.814**	0.842**	0.921**	0.935**	0.960**	0.954**	1.000									
Cr-Root	-0.642**	-0.790**	-0.762**	-0.725**	-0.757**	-0.802**	-0.828**	-0.828**	-0.845**	-0.841**	1.000								
Cr-Leaves	-0.565**	-0.707**	-0.653**	-0.646**	-0.721**	-0.747**	-0.732**	-0.750**	-0.783**	-0.755**	0.944**	1.000							
Cr-Stem	-0.584**	-0.712**	-0.648**	-0.653**	-0.701**	-0.737**	-0.703**	-0.761**	-0.804**	-0.716**	0.798**	0.831**	1.000						
Cr-Seed	-0.579**	-0.673**	-0.602**	-0.633**	-0.749**	-0.706**	-0.667**	-0.697**	-0.688**	-0.652**	0.867**	0.890**	0.777**	1.000					
NPPP	0.864**	0.975**	0.940**	0.862**	0.799**	0.836**	0.868**	0.936**	0.901**	0.885**	-0.683**	-0.572**	-0.594**	-0.540**	1.000				
NSPP	0.817**	0.828**	0.850**	0.764**	0.807**	0.804**	0.826**	0.844**	0.828**	0.850**	-0.650**	-0.579**	-0.560**	-0.464*	0.818**	1.000			
HSW	0.786**	0.824**	0.835**	0.869**	0.686**	0.787**	0.763**	0.819**	0.773**	0.783**	-0.608**	-0.416*	-0.482*	-0.419*	0.844**	0.799**	1.000		
SY18-19	0.854**	0.960**	0.952**	0.847**	0.773**	0.799**	0.857**	0.905**	0.870**	0.845**	-0.647**	-0.518**	-0.563**	-0.523**	0.985**	0.817**	0.879**	1.000	
SY19-20	0.869**	0.988**	0.966**	0.855**	0.824**	0.839**	0.887**	0.944**	0.927**	0.889**	-0.721**	-0.620**	-0.645**	-0.593**	0.989**	0.830**	0.839**	0.987**	1.000
(ii)																			
	PH	PFW	PDW	NPB	NSB	Chl-a	Chl-b	Total Chl	NR	NS	Cr-Root	Cr-Leaves	Cr-stem	Cr-Seed	NPPP	NSPP	HSW	SY18-19	SY19-20
PH	1.000																		
PFW	0.919**	1.000																	
PDW	0.921**	0.988**	1.000																
NPB	0.886**	0.957**	0.976**	1.000															
NSB	0.879**	0.956**	0.936**	0.862**	1.000														
Chl a	0.951**	0.887**	0.899**	0.864**	0.843**	1.000													
Chl b	0.899**	0.920**	0.907**	0.813**	0.941**	0.869**	1.000												
Total Chl	0.957**	0.975**	0.971**	0.937**	0.928**	0.964**	0.926**	1.000											
NR	0.903**	0.963**	0.938**	0.910**	0.917**	0.899**	0.911**	0.963**	1.000										
NS	0.925**	0.929**	0.907**	0.860**	0.907**	0.915**	0.928**	0.959**	0.950**	1.000									
Cr-Root	-0.689**	-0.752**	-0.745**	-0.713**	-0.646**	-0.773**	-0.749**	-0.791**	-0.842**	-0.765**	1.000								
Cr-Leaves	-0.553**	-0.616**	-0.633**	-0.590**	-0.517**	-0.612**	-0.653**	-0.632**	-0.652**	-0.541**	0.852**	1.000							
Cr-Stem	-0.622**	-0.652**	-0.650**	-0.598**	-0.585**	-0.689**	-0.642**	-0.694**	-0.726**	-0.672**	0.895**	0.813**	1.000						
Cr-Seed	-0.672**	-0.704**	-0.692**	-0.641**	-0.617**	-0.758**	-0.707**	-0.758**	-0.784**	-0.747**	0.962**	0.810**	0.956**	1.000					
NPPP	0.873**	0.981**	0.985**	0.973**	0.925**	0.823**	0.865**	0.930**	0.912**	0.867**	-0.680**	-0.582**	-0.583**	-0.617**	1.000				
NSPP	0.507*	0.584**	0.632**	0.713**	0.480*	0.581**	0.414*	0.597**	0.516**	0.452*	-0.485*	-0.457*	-0.341*	-0.416*	0.628**	1.000			
HSW	0.896**	0.891**	0.898**	0.829**	0.924**	0.910**	0.907**	0.922**	0.860**	0.903**	-0.619**	-0.501*	-0.566**	-0.615**	0.850**	0.440**	1.000		
SY18-19	0.760**	0.904**	0.932**	0.943**	0.838**	0.754**	0.729**	0.851**	0.814**	0.747**	-0.622**	-0.553**	-0.557**	-0.565**	0.955**	0.745**	0.770**	1.000	
SY19-20	0.885**	0.968**	0.982**	0.960**	0.936**	0.852**	0.892**	0.937**	0.917**	0.891**	-0.696**	-0.588**	-0.598**	-0.626**	0.982**	0.646**	0.866**	0.941**	1.000

PH = plant height; PFW = plant fresh weight; PDW = plant dry weight; NPB = number of primary branches; NSB = number of secondary branches; NR = nitrogen root; NS = nitrogen shoot; NPPP = number of pod/plants; NSPP = number of seed/plants; HSW = hundred seed weight; SY18-19 = seed yield 2018–19; SY19–20 = seed yield 2019–20. (P = ≤ 0.001)

** highly significant at 1% level;

* non-significant at 5% level.

## Discussion

Chromium toxicity has affected agricultural soil worldwide due to the uncontrolled discharge of hazardous Cr-containing effluents into the environment by industries. As a HM pollutant, Cr has a significantly negative impact on plants. Heavy Metals, especially Cr, induce alterations in numerous physiological processes and affect a myriad of biochemical pathways resulting in decreased or increased production of various metabolites during seed germination. These findings are in agreement with those of Kundu *et al*. [49]. In the present study, we investigated the effects of Cr toxicity on the seed germination, morpho-physiological characteristics, biochemical parameters, antioxidant functions, and enzymatic activity of two hydroponically-grown chickpea varieties. Cr toxicity affected seed germination by decreasing the germination percentage and vigour indices. A similar result was reported for seeds of *Hibiscus esculentus* L. [50], and *Cucumis melo* L. [51] germinated in different concentrations of Cr. Decreases in seed germination rates by 86% and 59% have also been reported under 1.5 mM and 1.8 mM Cr concentrations, respectively, in *Plantago ovata* Forsk [49], by 86.89% under 0.5 mM Cr concentration in *Oryza sativa* L. [52], by 63% under 100 ppm Cr in *T*. *aestivum* [13], by 48.88% under 100 mg/kg Cr in *H*. *esculentus* [50], and by 57% under 80 ppm Cr in *Saccharum* sp. [53]. In addition to the effects on seed germination, the effects of Cr on seedling growth parameters were also quite marked, since plant growth and biomass production decreased. At high Cr concentrations (120 μM), the Pusa 2085 variety exhibited greater root and shoot growth, greater biomass production, and higher seedling survival than the Pusa Green 112 variety. High Cr concentrations significantly affected the growth of the radicle in chickpea seedlings. Similar observations have been made by other researchers in *Basella alba* L. [54], Chinese cabbage [36], *Chrysopogon zizanioides* (L.) Roberty [55], *Oryza sativa* L. [56, 57], *V*. *radiata* (L.) R. Wilczek [29], *T*. *aestivum* [16, 18, 21], *B*. *napus* L. [14], and chickpea [34]. In a recent study, Cr also negatively affected the growth of plants by impairing essential metabolic processes [58]. Such molecular shifts in plant organs can lead to shunted plant growth and reduced biomass [14]. In the present study, the reduced root lengths under Cr toxicity may be due to the accumulation of Cr in roots, which damages the shoot ultrastructure. Cr treatments have also been reported to reduce root and shoot lengths, as well as the fresh and dry weights of various plant species, such as *O*. *sativa* [59] and *B*. *napus* [14]. Our results showed that MDA and H_2_O_2_ production and EL significantly increased with increasing Cr levels. The increase in ROS may be associated with the oxidative damage caused by Cr toxicity. Cr stress is known to generate ROS (H_2_O_2_ and superoxide) in excess and to cause lipid peroxidation. ROS in low concentrations act as a chemical messenger, regulating plant responses through hormones; however, at higher concentrations, they damage some of the essential macromolecules of cells, i.e., DNA, lipids, and proteins [60]. The lipid peroxidation caused by ROS damages biological membranes by mitochondrial respiratory chain breakage and renders the membranes vulnerable to oxidative damage. Lipid peroxidation, therefore, is considered a marker of oxidative stress in plants. MDA is a major product of lipid peroxidation, and thus it is also used as a marker for oxidative stress. In the present study, Cr-induced oxidative stress was observed in both chickpea varieties. However, it was more pronounced in Pusa Green 112 than in Pusa 2085, which suggested that Pusa 2085 is more tolerant to ROS production due to Cr toxicity. It has been observed in numerous studies that high Cr concentrations increased oxidative stress owing to the role of Cr in increasing the ROS, MDA, and EL contents of root and leaf tissues. This has been reported in various crops such as *B*. *napus* L. [61], *Zea mays* L. [20], *O*. *sativa* [57], and *Brassica oleracea* L. [11]. An increase in H_2_O_2_ levels was also observed in *O*. *sativa* seedlings subjected to Cr toxicity [59]. According to Dat *et al*. [62], at low levels, H_2_O_2_ acts as a messenger molecule and it is involved in various biochemical processes. Conversely, high levels of H_2_O_2_ production in Cr-treated roots and shoots triggers antioxidative enzyme activities, which suggests that membrane damage occurs due to the production and accumulation of excess ROS under Cr toxicity.

The protective role of proline varies according to species. As the Cr concentration increased to 120 μM, the production and accumulation of proline also increased rapidly in the seedlings of both chickpea varieties (Fig 6). The present results also indicated that Pusa 2085 had a higher capacity for proline accumulation in response to Cr stress than Pusa Green 112. This outcome was expected because proline is considered an energy source; its oxidative metabolism in the mitochondria helps driving oxidative phosphorylation and ATP synthesis in recovering tissues. Karthik *et al*. [63] reported similar findings of increased proline levels due to Cr toxicity in *P*. *vulgaris*.

An increase in antioxidant enzyme activities was also found in the leaves of both chickpea varieties with increasing Cr concentrations (Fig 7a–7e). Plants protect themselves against oxidative damage due to Cr stress by promoting the antioxidant activities of SOD, POD, CAT, APX, and GR [64]. In the present study, antioxidant enzyme activities varied with the different Cr concentrations. SOD and GR showed a pattern of increase in both chickpea species, whereas POD, APX, and CAT, showed an increase at low Cr concentration and a decline at high Cr concentrations (Fig 7a–7e). Although the activities of antioxidant enzymes in the leaves of Pusa 2085 were generally higher than that in the leaves of Pusa Green 112, a decrease was observed at higher concentrations of Cr, which might be due to severe oxidative stress, as previously described [17]. Previous studies have shown that APX activity decreased with increased metal toxicity in *Brassica juncea* (L.) [65], *B*. *napus* [66], and *T*. *Aestivum* [18]. Similar results have been observed in various plants subjected to Cr toxicity [11, 20, 67, 68].

In addition to plant growth, plant biomass, Chl *a*, Chl *b*, and total Chl contents, as well as other parameters, also decreased with increasing Cr concentrations in potted plants. Similarly, a significant decrease in photosynthetic pigments was found for *Catharanthus roseus* (L.) G.Don [69]. In the present study, the Chl content was lower in Pusa Green 112 than in Pusa 2085. Similar results have also been reported by Wani *et al*. [34]. Cr-induced reduction in photosynthetic pigments might be due to the alteration of the chloroplast ultrastructure and inhibition of gas exchange [23, 25, 70]. Many researchers have reported that Cr decreased the amount of Chl in *Pistia stratiotes* L. [64], chickpea [34], *Camellia sinensis* (L.) Kuntze [19], *Z*. *Mays* [71, 72], *V*. *radiata* [29], *Citrus limon* (L.) Osbeck and *Citrus reshni* hort. ex Tanaka [73], *P*. *ovate* [49], and *B*. *oleracea* [11]. Additionally, Kumar *et al*. [67] reported that Cr toxicity significantly reduced the Chl content of *Sorghum bicolor* (L.) Moench. The decrease in Chl content under Cr stress may be due to a reduction in the Chl biosynthesis enzyme, which is compromised under Cr stress [74, 75].

As indicated in the present study, the reduction in the nitrogen content of chickpea plants was possibly due to the decrease of chickpea–Mesorhizobium symbiosis, as indicated by the decline in nodulation. The yellowing of the leaves was attributed to nitrogen deficiency, which may be due to the reduction of Chl biosynthesis and to the effect of HMs on nitrogen-based targets [76]. Thus, increasing Cr concentration in soil seems to decrease the nitrogen content in the roots and shoots of plants. Similarly, Wani *et al*. [34] reported that the nitrogen content of chickpea roots and shoots was reduced owing to the toxic effect of HMs. In the present study, both positive and negative effects were observed. For example, a Cr concentration of 120 μM showed a negative effect on the nitrogen content of plant roots and shoots, whereas, at low Cr concentration (30 μM), it was increased in both varieties.

Protein content, which was influenced by Cr toxicity, is an important parameter in chickpea seeds. In the present study, the reduction in GP suggested that enzymes and functional proteins were affected by an increase in Cr concentrations. Additionally, the indirect effect of this HM on overall plant metabolism and possibly on their symbiotic partners may result in a decrease of GP due to a decrease in the availability of nitrogen. In general, high levels of Cr adversely affected growth and symbiosis, which resulted in reduced seed production in both varieties, although seed production increased in both varieties at low Cr levels (Fig 12a and 12b). In addition, a more significant reduction of GP was observed in Pusa Green 112 than in Pusa 2085. Decreases in growth attributes, yield, and yield parameters of both chickpea varieties were directly correlated with increased Cr concentrations (60–120 μM). Similarly, Wani *et al*. [77] reported that Cr (136 mg/kg in pot-grown conditions) decreased plant growth, biomass, grain yield, and other parameters compared to the control treatment. In addition, Bishnoi *et al*. [78] reported that Cr contamination in soil adversely affected the growth and yield parameters of *P*. *sativum* plants. Further, under Cr toxicity, seeds were not formed in some pods, and the seed number per pod was significantly reduced [78]. Sharma *et al*. [79] indicated that at a hexavalent Cr concentration of 1 mM the grain yield of *T*. *aestivum* was severely affected to the point of no grain formation. Similarly, Anjum *et al*. [20] reported that maize plant growth, yield, and yield-related parameters decreased under Cr stress. Sundaramoorthy *et al*. [56] reported that yield and seed production was reduced with high levels of Cr in irrigation water. In the present study, the chickpea plant growth parameters of 100-grain weight, seed yield, and number of pods per plant were decreased under high Cr concentrations, whereas during the 2018–19 and 2019–20 pot experiments, yield production per plant increased in both varieties at low Cr levels (Fig 8a–8d). The higher productivity of Pusa 2085 may be due to its slower uptake and lower Cr accumulation compared with Pusa Green 112. The reductions in yield in both chickpea varieties may thus be due to the different Cr treatments.

The accumulation of Cr in the leaves, stems, and roots at 95 DAS and in grains at 140 DAS differed between the various treatments. The fact that the Cr content in the roots was higher than that observed in the stem, leaves, and grains suggested that roots were the primary organ of Cr accumulation in both chickpea varieties. Kumar *et al*. [80] also reported that Cr accumulation was highest in the roots followed by the leaves and stems of *T*. *aestivum* plants under Cr stress. Additionally, many studies have reported that the highest Cr content was observed in the roots of *Hordeum vulgare* L. [81] and *B*. *napus* [14]. The higher Cr accumulation in the vegetative parts of Pusa Green 112 might be associated with its higher sensitivity to Cr stress, whereas the low accumulation of Cr in the Pusa 2085 genotype might be associated with its higher tolerance. Shankar *et al*. [82] observed the translocation of Cr from root to shoot in comparatively tolerant plants, which were considered root accumulators. Cr toxicity in plant systems and the modulation of physiological mechanisms depends mainly on the quantity of Cr accumulated, its mobilisation, and its final deposition in various plant tissues [18, 83]. Our results are also in agreement with Anjum *et al*. [20], who demonstrated that the absorption, translocation, and deposition of Cr varies among the different plant organs, with roots exhibiting the highest content. Our results showed that the Cr content of Pusa 2085 seeds was lower than that of Pusa Green 112 (Fig 15a and 15b), suggesting less translocation of Cr from other vegetative parts to the seeds in this variety. Due to the high grain productivity of Pusa 2085, the dilution effect of the Cr content in seeds cannot be ignored when considering the low Cr content of the other plant organs. The higher concentration of Cr in all parts of the plant may have resulted in its decreased growth attributes, biomass, Chl content, and yield attributes.

## Conclusion

The extensive use of Cr in different manufacturing industries, which has increased over the years, has resulted in severe environmental contamination. The severe Cr pollution adversely affects crops including chickpea. Exposure to Cr also has negative impacts on the seed germination, and the morpho-physiological and biochemical characteristics, including plant growth, Chl content, N content, and GP of chickpea. The adverse effects seem to be a consequence of increased Cr bioaccumulation, which causes heavy metal stress. Particularly, Cr stress enhances MDA and H_2_O_2_ production, and EL, while reducing total protein contents in chickpea seeds. In addition, Pusa 2085 exhibited greater tolerance to Cr toxicity than Pusa Green 112. However, for a comprehensive understanding of the mechanisms of tolerance of Cr toxicity, interactions among the above processes should be explored extensively at the genetic level across different crops. Such studies could facilitate the management of heavy metal-induced stress in crops, which is increasingly becoming a major challenge in agricultural systems.

## Supporting information

S1 Table Physicochemical properties of soil used for pot experiments. (DOCX)

S2 Table Effects of different chromium (Cr) concentrations (0, 30, 60, 90, and 120 μM) on germination and germination index of two chickpea varieties. (DOCX)

S1 Fig Vigour index of two chickpea varieties under the different chromium (Cr) treatments. (TIF)

S2 Fig Effects of the various chromium (Cr) on root and shoot nitrogen contents of two chickpea varieties grown in pots 95 days after treatment (DAT). (a) Pusa 2085 (b) Pusa Green 112.
(TIF)

S3 Fig Effects of various levels of chromium (Cr) on the protein contents of seeds of two chickpea varieties grown in pots (Pusa 2085 and Pusa Green 112) 140 days after treatment (DAT). (TIF)
