# Supplementary material for: Effect of chromium (VI) toxicity on morpho-physiological characteristics, yield, and yield components of two chickpea (Cicer arietinum L.) varieties
Source: PLoS One. 2020 Dec 3;15(12):e0243032. doi: 10.1371/journal.pone.0243032 (PMC7714171; doi:10.1371/journal.pone.0243032)
Supplement: S1 Table — (DOCX) [file pone.0243032.s001.docx]

S1 Table. Physicochemical properties of soil used for pot experiment

| Properties | Sandy loam Soil |
| --- | --- |
| Sand (%) | 56.00 |
| Silt (%) | 20.00 |
| Clay (%) | 24.00 |
| Organic matter (%) | 0.37 |
| Phosphorous (P) mg kg ⁻^1^ | 21.40 |
| Potassium (K) mg kg ⁻^1^ | 176.0 |
| Iron (Fe) mg kg ⁻^1^ | 1.24 |
| Nitrogen (N) mg kg ⁻^1^ | 6.54 |
| Electrical conductivity (dsm⁻^1^) | 2.60 |
| Calcium carbonate (CaCO_3_)mg kg ⁻^1^ | 1.54 |
| Copper (Cu)mg kg ⁻^1^ | 0.002 |
| Manganese (Mn)mg kg ⁻^1^ | 0.045 |
| Zinc (Zn) mg kg ⁻^1^ | 0.07 |
| Chromium (Cr) | BDL |
| Lead (Pb) | BDL |
| Cadmium (Cd)mg kg ⁻^1^ | 0.001 |
| pH | 7.31 |
| Aluminum (Al)mg kg ⁻^1^ | 0.004 |

Note: -BDL- Below Detection Limit
