# Supplementary material for: Effect of chromium (VI) toxicity on morpho-physiological characteristics, yield, and yield components of two chickpea (Cicer arietinum L.) varieties
Source: PLoS One. 2020 Dec 3;15(12):e0243032. doi: 10.1371/journal.pone.0243032 (PMC7714171; doi:10.1371/journal.pone.0243032)
Supplement: S2 Table — (DOCX) [file pone.0243032.s002.docx]

| S2 Table. Effect of different treatment concentrations of chromium (0, 30, 60, 90 and 120 µM) on germination and germination index of two chickpea seeds | | | | | | | | |
| --- | --- | --- | --- | --- | --- | --- | --- | --- |
| Treatment  (µM) levels | Seed germination (percentage) | | | | Germination index (percentage) | | | |
|  | Pusa 2085 | Variation | Pusa Green 112 | Variation | Pusa 2085 | Variation | Pusa Green 112 | Variation |
| 0 | 97.47±0.22^a^ | $-$ | 98.59±0.41^a^ | $-$ | 57.50±0.19^a^ | $-$ | 57.46±0.19^a^ | $-$ |
| 30 | 97.88±0.25^a^ | $+$0.41 | 93.28±0.26^b^ | $-$5.31 | 58.24±0.20^a^ | $+$0.74 | 53.37±0.34^b^ | $-$4.09 |
| 60 | 82.73±2.06^b^ | $-$14.74 | 67.48±0.69^c^ | $-$31.11 | 51.01±1.10^b^ | $-$6.49 | 35.73±0.95^c^ | $-$21.73 |
| 90 | 54.65±1.19^c^ | $-$42.82 | 50.85±0.57^d^ | $-$47.74 | 33.88±0.50^c^ | $-$23.62 | 32.70±0.24^d^ | $-$24.76 |
| 120 | 44.34±0.64^d^ | $-$53.13 | 40.79±0.49^e^ | $-$57.80 | 25.87±0.45^d^ | $-31.63$ | 24.71±0.35^e^ | $-$32.75 |
| LSD_0.05_ | 6.08 |  | 2.77 |  | 3.24 |  | 2.73 |  |
| Data shown are mean values of five replicates with standard error (S.E.). mean values in the same column followed by same letters do not statistical differences at p < 0.05 according to the ANOVAs. | | | | | | | | |
