# Supplementary figures and images for: Effect of chromium (VI) toxicity on morpho-physiological characteristics, yield, and yield components of two chickpea (Cicer arietinum L.) varieties
Source: PLoS One. 2020 Dec 3;15(12):e0243032. doi: 10.1371/journal.pone.0243032 (PMC7714171; doi:10.1371/journal.pone.0243032)

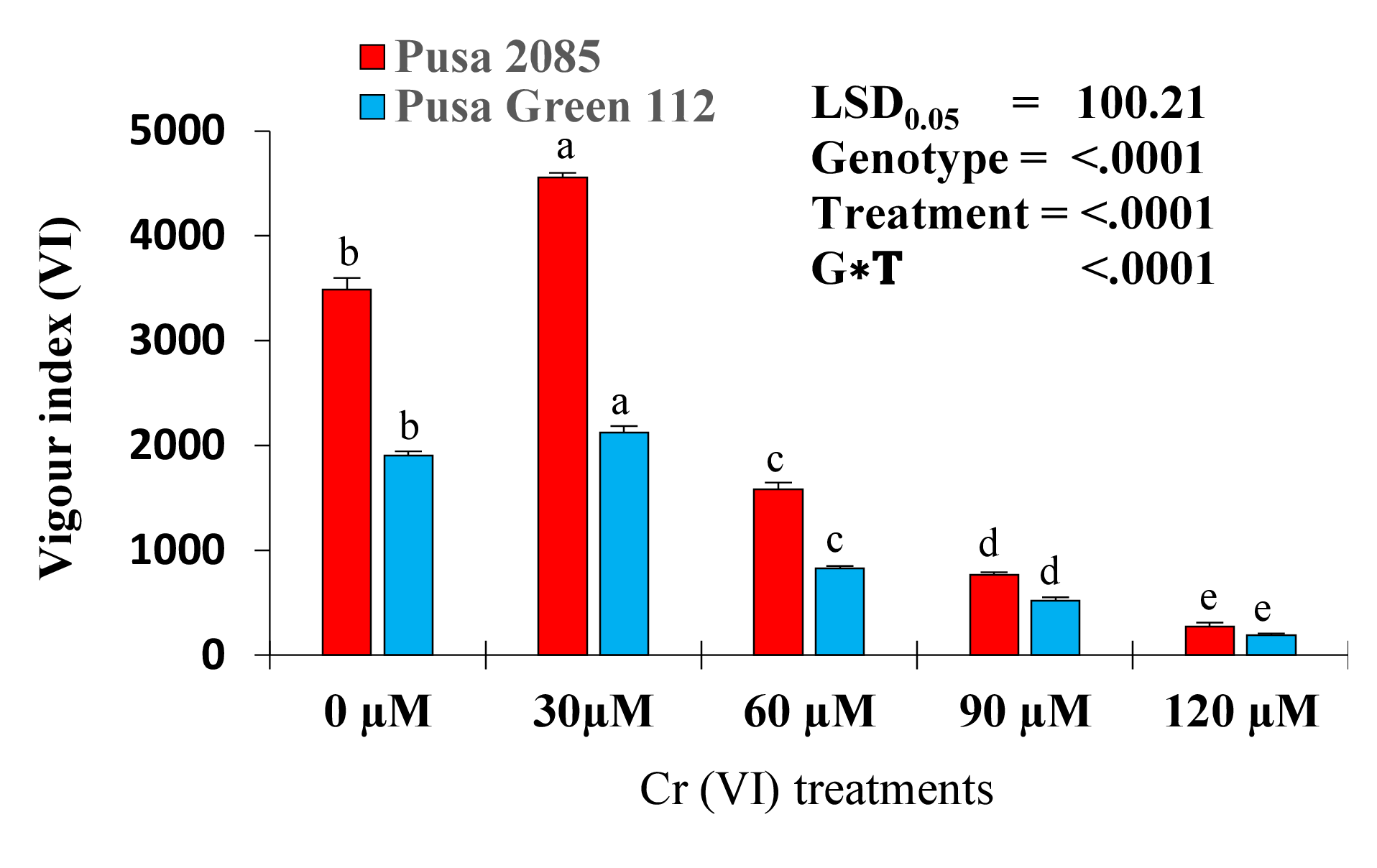

Supplement: S1 Fig — (TIF) [file pone.0243032.s003.tif]

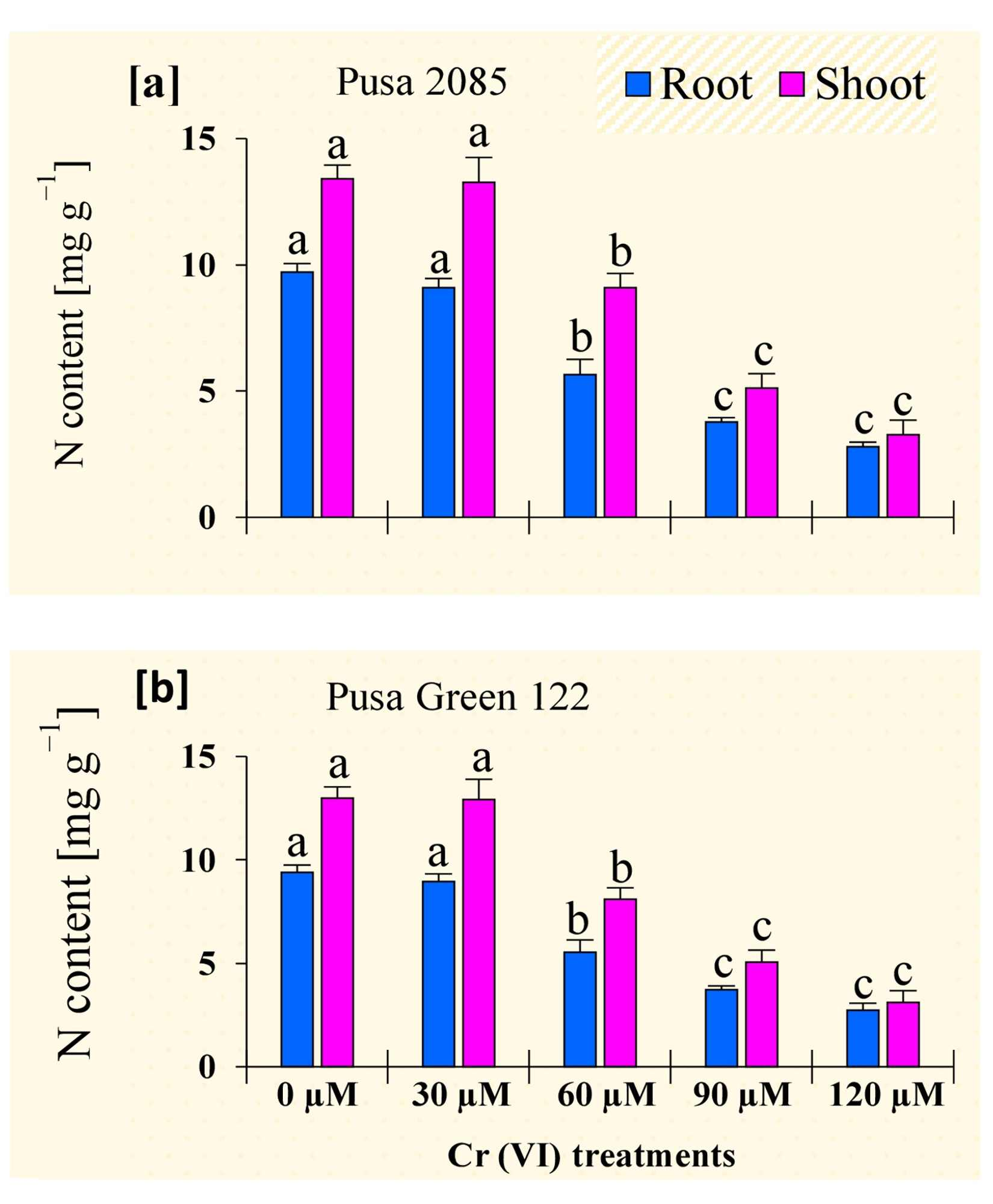

Supplement: S2 Fig — (a) Pusa 2085 (b) Pusa Green 112. (TIF) [file pone.0243032.s004.tif]

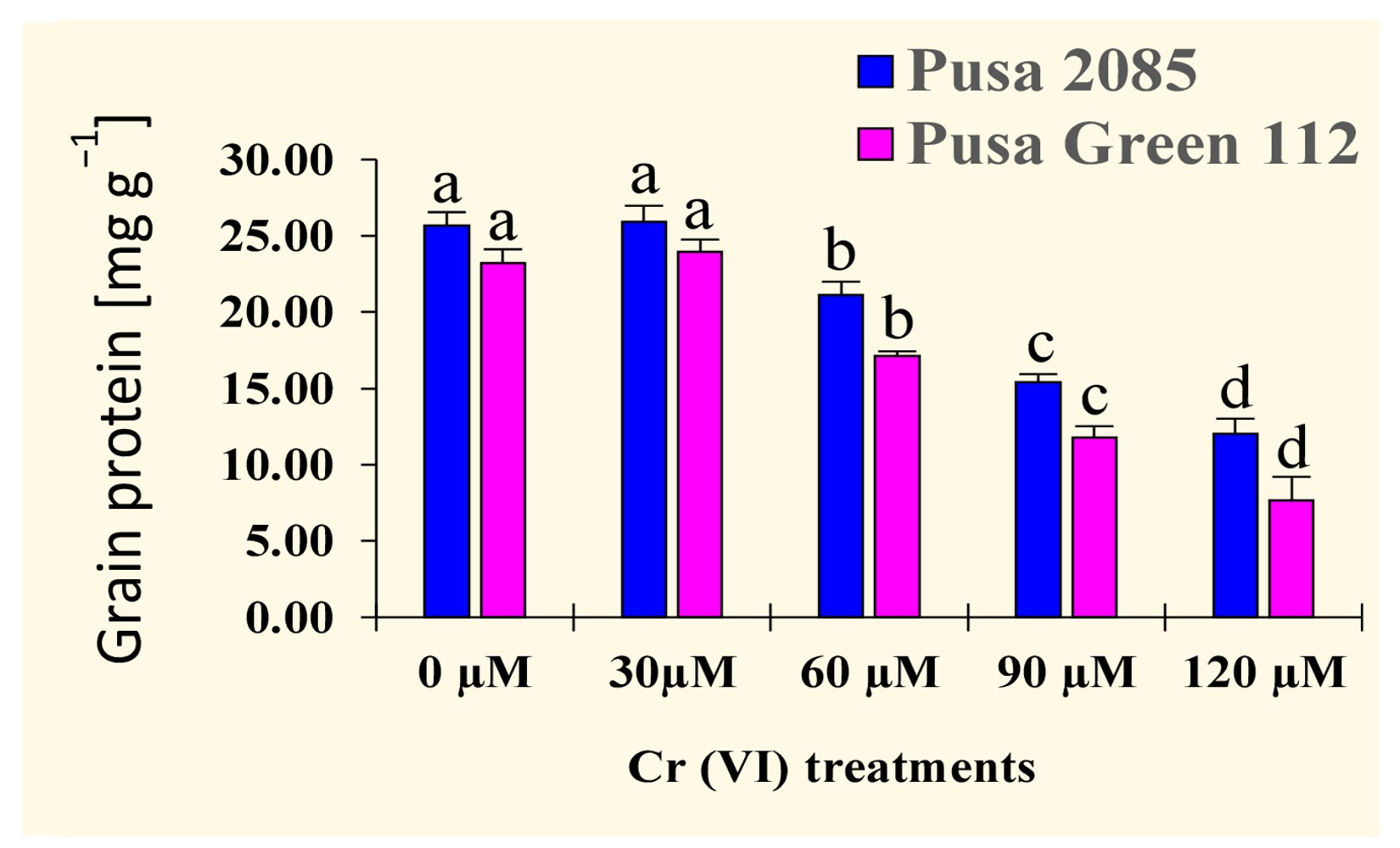

Supplement: S3 Fig — (TIF) [file pone.0243032.s005.tif]
